# The origin of the expressed retrotransposed gene *ACTBL2* and its influence on human melanoma cells’ motility and focal adhesion formation

**DOI:** 10.1038/s41598-021-82074-x

**Published:** 2021-02-08

**Authors:** Natalia Malek, Aleksandra Michrowska, Ewa Mazurkiewicz, Ewa Mrówczyńska, Paweł Mackiewicz, Antonina J. Mazur

**Affiliations:** 1 Department of Cell Pathology, Faculty of Biotechnology, University of Wroclaw, ul. Joliot-Curie 14a, 50-383 Wroclaw, Poland; 2 Department of Bioinformatics and Genomics, Faculty of Biotechnology, University of Wroclaw, ul. Joliot-Curie 14a, Wroclaw, 50-383 Poland

**Keywords:** Cell adhesion, Cell migration, Cytoskeleton, Phylogeny

## Abstract

We have recently found that β-actin-like protein 2 (actbl2) forms complexes with gelsolin in human melanoma cells and can polymerize. Phylogenetic and bioinformatic analyses showed that actbl2 has a common origin with two non-muscle actins, which share a separate history from the muscle actins. The actin groups’ divergence started at the beginning of vertebrate evolution, and actbl2 actins are characterized by the largest number of non-conserved amino acid substitutions of all actins. We also discovered that *ACTBL2* is expressed at a very low level in several melanoma cell lines, but a small subset of cells exhibited a high *ACTBL2* expression. We found that clones with knocked-out *ACTBL2* (CR-*ACTBL2*) or overexpressing actbl2 (OE-*ACTBL2*) differ from control cells in the invasion, focal adhesion formation, and actin polymerization ratio, as well as in the formation of lamellipodia and stress fibers. Thus, we postulate that actbl2 is the seventh actin isoform and is essential for cell motility.

## Introduction

Actins are a group of very well-characterized proteins crucial for many cellular processes, including cell motility, maintenance of cell shape, chemoattractant migration, trafficking of cellular organelles and chromosomes, mitosis, muscle contraction, transcription, and DNA repair^1–3^. Until now, it has been believed that mammals have only six actin isoforms divided into two classes: class I (non-muscle) [β-non-muscle (human gene *ACTB*) and γ-non-muscle actin (*ACTG1*)] and class II (muscle) [α-skeletal muscle (*ACTA1*), α-smooth muscle (*ACTA2*), α-cardiac (*ACTC1*), and γ-smooth muscle (*ACTG2*)]^4–6^. β-actin differs from the non-muscle γ isoform only in a few amino acid residues within the first ten amino acids at N-terminus. Although β- and γ-non-muscle actins are expressed mainly in non-muscle cells, the expression levels of their genes (*ACTB* and *ACTG1*) differ among tissue types. There is much interest in the expression patterns of *ACTB* and *ACTG1* under pathological conditions, such as tumors, reparative processes, and cardiovascular diseases^7^. The functional diversification of non-muscle isoactins and their subcellular localization is still under active research. Recently, we reported that β- and γ-actin are non-redundant regarding their involvement in 2D and 3D motility and focal adhesion formation of human melanoma cells^8^.

In one of our earlier studies^9^, we obtained data showing that gelsolin, one of the many actin-binding proteins (ABPs), interacts specifically with β-actin-like protein 2 (actbl2) in human melanoma cells. *ACTBL2*, the human gene encoding actbl2 (Uniprot accession number Q562R1), is not a pseudogene but a retrotransposed gene because it does not contain introns. This gene (Gene Id: 345651), located on chromosome 5 (5q11.2), is transcribed, and its mRNA is translated. The Q562R1 record is associated with an article^10^ describing κ-actin transcripts as a new actin family found in hepatocellular carcinoma. However, a detailed analysis of the nucleotide sequences mentioned in this article points not at *ACTBL2* but POTE-actin genes^11^. We have already shown that actbl2 could polymerize, as revealed by the analysis of A375 melanoma cells ectopically expressing HA-actbl2 (hemagglutinin-tagged actbl2)^9,12^. F-actin with incorporated HA-actbl2 was present in lamellipodia, filopodia, and invadopodia structures playing crucial roles in cell migration and invasion^13^. Until now, only one study has addressed the functional role of actbl2. Hoedebeck et al.^14^ have shown that the silencing of *ACTBL2* leads to diminished motility of human arterial smooth muscle cells. These authors also demonstrated that the expression of *ACTBL2* in smooth muscle cells under stretch conditions depends on the nuclear factor 5 of activated T-cells (NFAT5).

Because there is scant published data concerning actbl2, we decided to investigate its role in human melanoma cells in terms of their 2D and 3D motility and ability to form focal adhesions. We obtained stable A375 clones, either devoid of actbl2 or overexpressing actbl2. We chose these cells as they are among the best-studied human melanoma cells.

The results generated using these clones clearly showed that manipulation of actbl2 expression has a clear impact on actin cytoskeleton organization, migration, invasion, and focal adhesion formation. Moreover, we conducted comprehensive phylogenetic and bioinformatic studies to reveal the origin of this expressed retrotransposed gene *ACTBL2*. We found that *ACTBL2* had a common ancestor with two other non-muscle actin isoforms, and sequences most similar to *ACTBL2* are present in marsupials and placental mammals, but sequences showing similarity to them can also be found in earlier diverged vertebrate lineages up to cartilaginous fishes. The presented data imply that actbl2, though expressed at very low levels, might be regarded as an additional actin isoform.

## Results

### Inferring phylogenetic relationships between actins including actbl2

**Figure 1:**
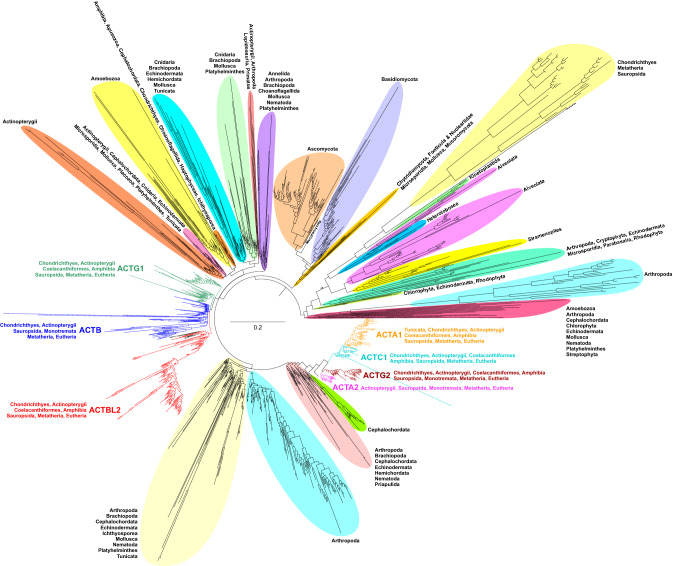
Phylogram obtained in MrBayes showing relationships between 1441 amino acid sequences of actins and their relatives. Seven groups of actins similar to human sequences: *ACTB*, *ACTG1*, *ACTA2*, *ACTA1*, *ACTC1*, *ACTG2*, and *ACTBL2* are indicated in different colors. Affiliation to major taxonomic groups is also shown for main clades.

Searches for sequences similar to actbl2 provided 13,694 amino acid sequences. Therefore, to comprehend such a massive set of sequences, we carried out their clustering in CLANS based on the results of pairwise BLASTP searches (Fig. S1). The analysis enabled us to distinguish clear clusters containing closely related actbl2 sequences, which were subjected to further studies. Phylogenetic trees obtained by different methods gave similar topologies. Figure 1 presents the general phylogram inferred in MrBayes for amino acid alignment. In turn, the Supplementary Figs. S2 and S3 include detailed cladograms with the taxonomic affiliation of individual sequences, as well as support values calculated in MrBayes and IQ-TREE for the approach based on amino acid sequences. We referred in the description to the seven actin groups according to their human gene names: *ACTB* (β-non-muscle actin), *ACTG1* (γ-non-muscle actin), *ACTA2* (α-smooth muscle actin), *ACTA1* (α-skeletal muscle actin), *ACTC1* (α-cardiac muscle actin), *ACTG2* (γ-smooth muscle actin), and *ACTBL2* (β-actin-like protein 2).

The most similar sequences to human *ACTBL2* created a distinct clade, which was grouped with *ACTG1* and the next *ACTB* homologs in IQ-TREE (Fig. S2). The whole clade consisting of three non-muscle (cytoplasmic) actin groups received quite high support (93%) in SH-aLRT. In the MrBayes tree, the relationships between these three groups were not resolved, but these groups were also clustered together (Fig. S3). Four other muscle actin groups created a separate clade (Fig. 1) supported in 92% by aLRT and 100% by bootstrap percentage (BP) (Fig. S2), as well as by 0.73 posterior probability (PP) in the Bayesian tree (Fig. S3). *ACTA1* and *ACTC1* homologs were more closely related in two trees with 85% aLRT support and 0.68 PP. They were clustered with *ACTA2*, and these three actins were grouped with *ACTG2* in IQ-TREE (Fig. S2), whereas in MrBayes, *ACTA2* and *ACTG2* were connected (Fig. S3).

Human *ACTBL2* was significantly (aLRT: 100%, BP: 100%, PP: 0.98) clustered with sequences from other mammals, placentals, and marsupials (Figs. S2 and S3). In the MrBayes tree, these mammalian sequences were additionally grouped with those from monotremes and all other main groups of vertebrates: birds, reptiles, amphibians, as well as cartilaginous and bony fishes (Fig. S3). The similarity of sequences from the diverse taxonomic groups to *ACTBL2* is supported by sensitive classification of sequences to actin groups using profile HMMs. Other actin groups, such as *ACTG1*, *ACTA1*, *ACTC1*, and *ACTG2*, were also found in the main groups of vertebrates. *ACTB* did not reveal homologs in amphibians, nor did *ACTA2* in cartilaginous fishes. However, the *ACTA1* group also included sequences from tunicates, a sister group to vertebrates. Interestingly, among *ACTB* homologs, several other human sequences were placed, which were annotated as POTE ankyrin domain family member E, F, I, and J^15^. This suggests that they could evolve from the β-non-muscle actins.

Two main actin classes, non-muscle and muscle, were separated in the trees based on amino acid sequences (Figs. 1, S2, and S3), and each of them was grouped with sequences assigned mainly to various groups of invertebrates as well as eukaryotic lineages other than Animalia (Metazoa). This indicates a separate origin and evolution for these actin classes in vertebrates. Interestingly, among the clades located outside the main actin groups, there are also members of vertebrates. These likely represent some actin-like proteins, which were also subjected to an independent evolution. The grouping of sequences from unrelated taxonomic lineages probably results from a high divergence rate, horizontal gene transfer or contamination, and incorrect sequence assignment to species. For example, we found several sequences assigned to bacteria identical or very similar to those from eukaryotes. Similar to that, some single eukaryotic sequences clustered within taxonomically distant eukaryotic lineages.

Trees based on the nucleotide sequences (Figs. S4 and S5) were poorer resolved, and many sequences assigned to individual actin groups (except for *ACTBL2* and *ACTG1*) were separated into several clades, not forming one monophyletic group. Especially, muscle actin sequences from bony fishes assigned to different actin groups were mixed in two phylogenetic trees and located basal to other members of actin groups, including higher vertebrates (tetrapods). However, the individual clades were highly supported, and the relationships at lower taxonomic levels were well resolved. The separation of actins into non-muscle and muscle classes was still clearly visible. The clade of muscle actins obtained 100% aLRT support, and most non-muscle actins were clustered with 85% or 99% aLRT support (Fig. S4). In contrast to the phylogenetic trees based on amino acid sequences, these two actin classes were not separated by other types of sequences but were clustered together. This grouping obtained 99% aLRT support but no significant support in the Bayesian approach.

Sequences similar to actbl2 created a monophyletic group in two trees based on the nucleotide sequences. In IQ-TREE, this group received 95% aLRT support and included representatives of amniotes and bony fishes (Fig. S4). In contrast, in the MrBayes tree, it included members of amniotes, coelacanths, and cartilaginous fishes (Fig. S5). The *ACTBL2* group was placed inside the leading group of *ACTB* with 99% aLRT support in IQ-TREE (Fig. S4). Most sequences of *ACTA2* and *ACTG2* were also clustered with 99% aLRT support in IQ-TREE (Fig. S4), whereas in the MrBayes tree, the *ACTA2* group was connected with *ACTC1* but without significant support (Fig. S5). In both trees, *ACTG1* homologs were located inside the *ACTB* clade.

### Individual actin groups show variable evolutionary rates

**Figure 2:**
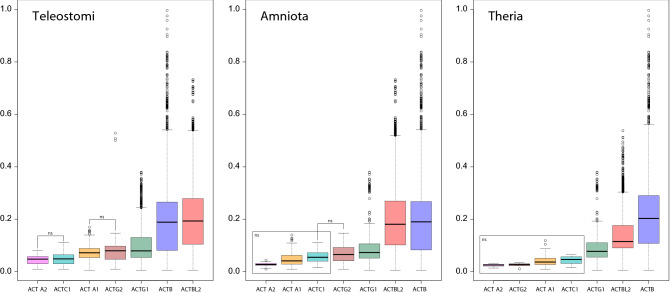
Box-plots of phylogenetic distances measured by the number of amino acid substitutions per site (y-axis) for seven actin groups (x-axis). Teleostomi includes bony fishes and tetrapods, Amniota contains reptiles, birds, and mammals, and Theria comprises marsupials and placentals. The thick line indicates the median. The colored box shows the quartile range, and the whiskers denote the range without outliers. All pairwise comparisons except those noted by ns (non-significant) are statistically significant with *p* < 0.05.

To study the evolutionary rate of particular groups of actin, we compared pairwise distances (measured by the number of amino acid substitutions per site) within each group received from the MrBayes tree based on the amino acid alignment (Fig. 2). To compare data representing the same evolutionary times, we calculated the distances for actin sequences from the same taxonomic groups. We considered three such groups: Teleostomi (bony fishes and tetrapods), Amniota (reptiles, birds, and mammals), and Theria (marsupials and placentals) (Fig. 2). *ACTBL2* and *ACTB* actins were characterized by a significantly higher divergence than other groups, i.e., 2.4–4–, 2.5–6.9–, and 1.5–7.8-times higher, according to medians, for the Teleostomi, Amniota, and Theria sets, respectively. The other non-muscle actin group, *ACTG1*, also showed significantly higher divergence (up to 3-times) than muscle actin groups, which were characterized by the lowest and homogenous substitution rate. The differences between phylogenetic distances of muscle actin groups were, in most cases, not statistically significant (Fig. 2).

### Unique amino acid substitutions can distinguish individual actin groups

**Figure 3:**
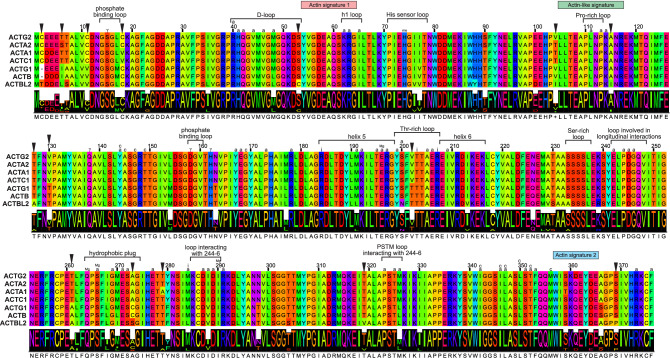
The alignment of the majority-rule consensus sequence for seven actin groups obtained from profile HMMs. Black triangles indicate columns, including non-conserved amino acid substitutions. Residues interacting with other residues of the actin filament were marked by a, and γ phosphate of nucleotide by γ. Residues co-ordinating inorganic phosphate ions were marked by i, and Mg^2+^ ions by Mg. Residues involved in the lower cleft were marked by c. Moreover, selected structural features of actins were marked, as well as actin signature 1 (PS00406), actin signature 2 (PS00432), and actin-like signature (PS01132). Annotation of residues was taken from^16,17^.

We assigned individual sequences to one of seven actin groups using profile hidden Markov models (HMMs) for seven groups individual of actins (Table S1). We presented sequence variation within and between these groups in Figs. 3 and S6. Out of 376 alignment positions, 321 are identical. Ninety-eight of them include residues involved in interacting with other residues in the actin filament, other proteins (myosin, tropomyosin, and nebulin), and nucleotide phosphate, as well as co-ordinating inorganic phosphate and Mg^2+^ ions (Fig. 3). Thirty-two alignment positions include at least one conserved substitutions, i.e., the substituted amino acid residues are physicochemically similar; they have assigned a positive score in the BLOSUM62 matrix (Figs. 3 and S6). Residues in 14 of these sites interact with residues of other proteins and cofactors (Fig. 3). At eight sites, the substituted residues do not differ in physicochemical properties and have been assigned 0 scores in the BLOSUM62 matrix. Residues of α-skeletal muscle actin in three of these sites participate in interactions with nebulin (Fig. 3).

However, the most interesting are non-conserved amino acid substitutions, i.e., involving amino acid residues characterized by different physicochemical properties and showing negative scores in the BLOSUM62 matrix (Figs. 3 and S6). We found 15 such sites in the alignment of profile HMMs. In six alignment sites (amino acid residue number 6, 11, 104, 130, 202, and 261 in Figs. 3 and S6), each of two actin classes, non-muscle and muscle, share the same or physicochemically similar residues, which differ between these classes, e.g., in hydrophobicity or polarity (Ile/Leu/Val/Ala vs. Ser/Thr) as well as aliphaticity/hydrophobicity and sulfur presence (Val vs. Cys). The consensus sequence of the muscle actins has Cys (more hydrophobic) and Thr (more hydrophilic) as the residue 2, which corresponds to deletion in the consensus alignment in *ACTB* and *ACTG1* actins. The N-terminal residues Met and Cys are post-translationally modified and removed in the muscle actins. In contrast, only the initial Met is processed and removed in the non-muscle actins^18^. This suggests that also the Thr residue in *ACTBL2* actin can be modified, e.g., phosphorylated. However, at this moment, there is no evidence supporting this.

Interestingly, *ACTBL2* actins showed the most significant number of unique non-conserved substitutions. At sites 53, 115, 127, 319, and 369, all other actin groups have the same residue in their consensus sequences, which are characterized by physicochemical features other than those in the *ACTBL2* consensus (Figs. 3 and S6). These residues differ in polarity and sulfur presence (Ser and Cys), aliphaticity and size (Ala and Ile), polarity and size (Thr and Ala), hydrophobicity and polarity (Thr and Val), as well as polarity and secondary structure propensity (Ser and Pro). Additionally, in the 273rd site located in the hydrophobic plug, *ACTBL2* actins have polar Ser in their consensus, whereas other sequences have non-polar Ala or Cys (Fig. 3). The substitution in the 53rd site is located inside D-loop. In *ACTBL2* actins, there are also other interesting, unique substitutions (Fig. 3); one generated hydroxylated residue Tyr from basic His (at the 88th alignment site) and the other Asn from hydroxylated Ser (at the 200th site). Many non-conserved changes in the *ACTBL2* sequence consensus are visible in the long branch leading to this actin in the phylogenetic tree based on the consensus sequences (Fig. S7). *ACTA1* actins have only one unique site (279) with a non-conserved substitution (non-polar Ala vs. polar Thr) (Figs. 3 and S6). Moreover, *ACTA1* and *ACTC1* actins share hydrophobic and large Val at the 18^th^ site of their consensus, whereas other actins possess tiny and sulfur-containing Cys.

### Comparison of phylogenetic relationships between actin groups obtained by various approaches

**Figure 4:**
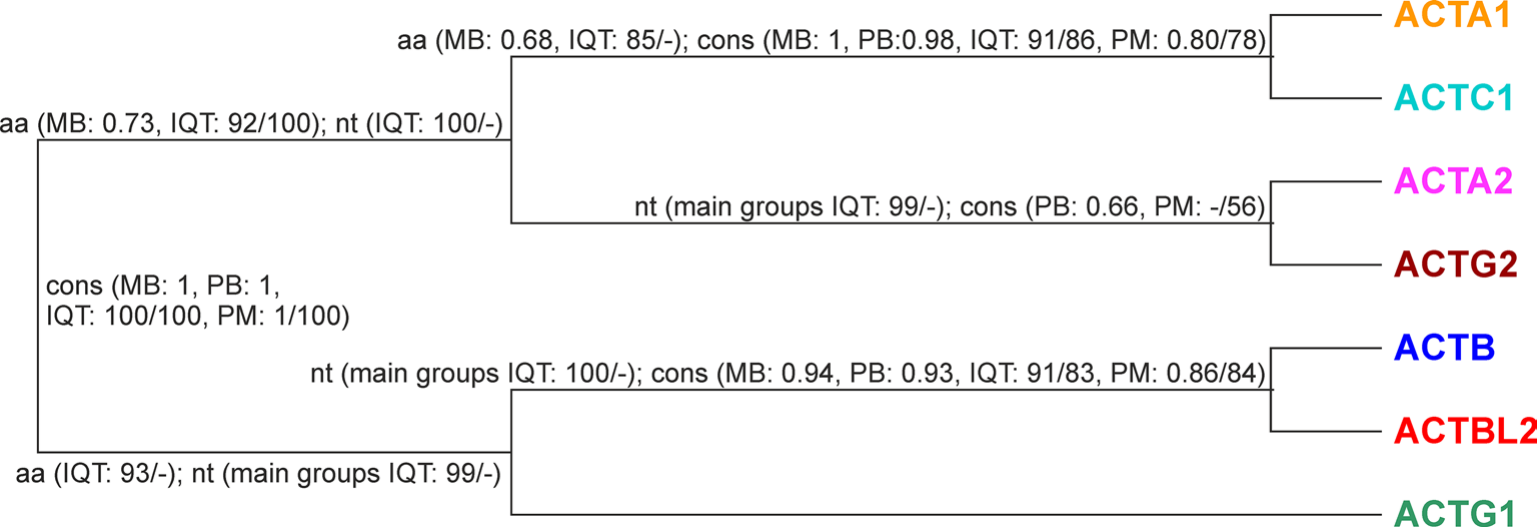
The most probable actin tree topology with support values obtained for various data sets: consensus sequences (cons), the full alignment of 1441 amino acid sequences (aa) and 3554 nucleotide sequences (nt), and by various software: IQ-TREE (IQT), morePhyML (PM), MrBayes (MB) and PhyloBayes (PB). Numbers at nodes for MB and PB correspond to posterior probabilities, whereas for IQT and PM to support values calculated by aLRT based on a Shimodaira-Hasegawa-like procedure (before the slash) and bootstrap analysis (after the slash). The support values lower than 50 are indicated by a dash “–”. The only clade in a competitive topology grouping *ACTBL2* with *ACTG1* was poorly supported in IQT tree: 84/-.

Phylogenetic analyses of amino acid profile HMMs and consensus sequences provided three topologies, which were gathered in Supplemental Fig. S8 together with those obtained for the full alignment of 1441 amino acid sequences. All trees separated non-muscle actins from muscle actins, as well as clustered *ACTA1* and *ACTC1* (striated muscle actins). Most trees (five of eight) joined clades *ACTA1* + *ACTC1* and *ACTA2* + *ACTG2* (smooth muscle actins) together. *ACTBL2* and *ACTB* were grouped in all four trees based on consensus sequences, whereas in trees obtained for profiles, *ACTB* was joined with *ACTG1*, and *ACTBL2* with *ACTG1* in IQ-TREE tree based on the full alignment (Fig. S8). Based on testing of the different tree topologies (Table S2) and including support values of individual clades, we can conclude that the topology presented in Figs. 4 and S8 as t1 is the most probable.

### Actbl2 is detected in several mass spectrometry studies proving its presence in normal and tumor cells

We searched the literature to determine whether actbl2 is indeed detected at the protein level in proteomic studies. We found several papers in which peptides unique only for actbl2 were detected (Fig. S9, Table S3)^19–39,41–43^. Some of the studies listed in Table S3 concern the ubiquitination of actbl2^24,26,28,29^. One study showed that actbl2 is phosphorylated^19^. These findings suggest that actbl2 undergoes similar post-translational modifications as other actin isoforms^40^. Actbl2 unique peptides were detected both in healthy tissues^23,33,36,41^ or cell lines^21,34,38^, and in tumor tissues^30–32,39,42^ or cell lines^21,22,24,26,28,29,35,43^.

### Actbl2—like β-, and γ-actin—is incorporated into F-actin structures, but is expressed at very low levels in melanoma cells

**Figure 5:**
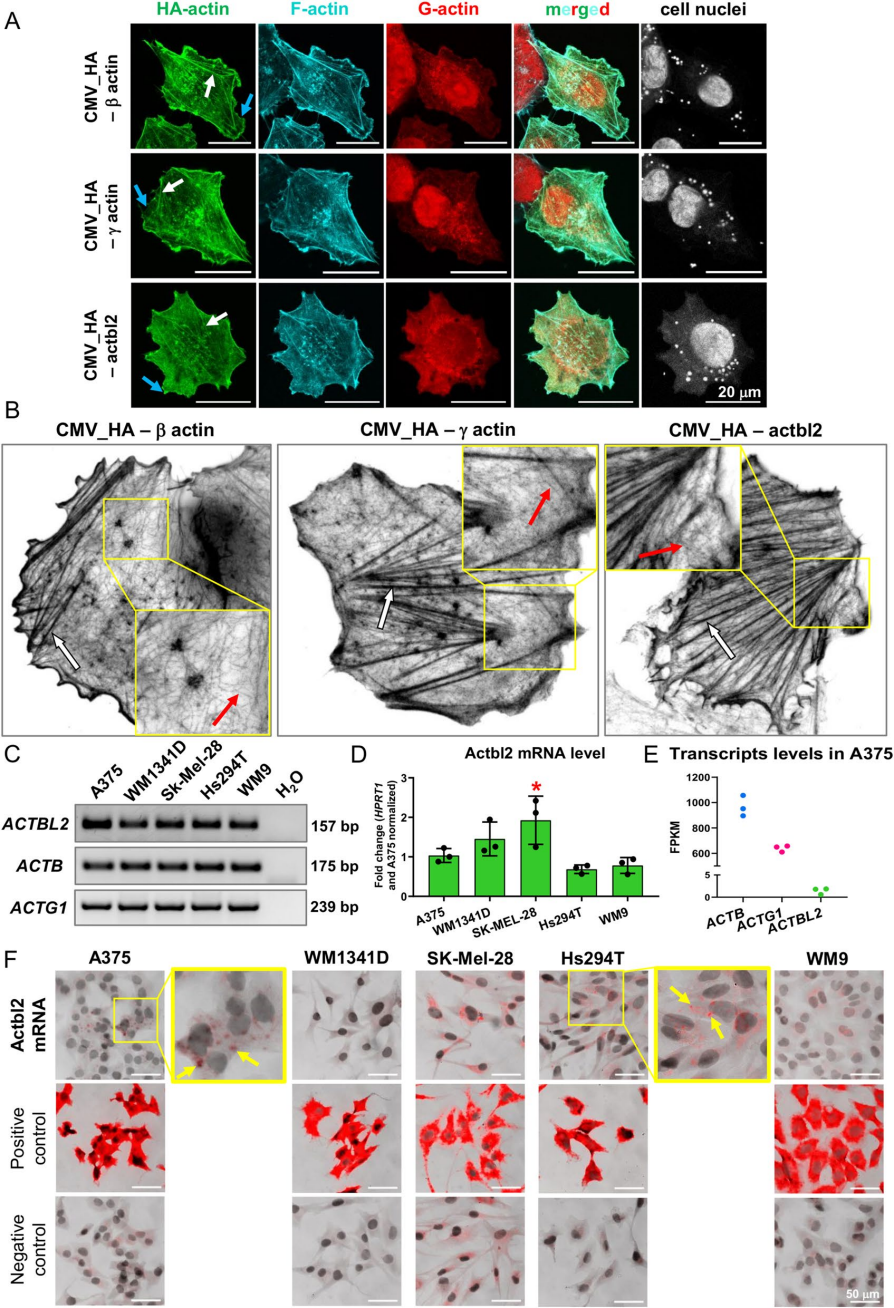
Actbl2 has incorporated into the same F-actin structures as β- and γ-actin, but its expression level in melanoma cell lines is very low. (**A**,**B**) A375 cells were transfected with plasmids coding for HA-β actin, HA-γ actin, and HA-actbl2. 24 h later, the cells were fixed and stained with anti-HA antibodies, phalloidin CruzFluor 350, DNase I Alexa Fluor 594, and DRAQ5 to detect HA-actins, F-actin, G-actin and nucleus, respectively (**A**). Alternatively, the cells were stained solely with anti-HA antibodies to detect HA-actins (**B**). Next, the cells were examined by confocal (**A**) and STED (**B**) microscopy. White arrows highlight stress fibers, blue arrows lamellipodium, and red arrows point at single actin filaments. (**C**) Analysis of PCR products of reactions, where cDNAs of five melanoma cell lines served as templates and primers recognizing coding sequences for β-actin, γ-actin, and actbl2 were used. 2% TAE-agarose gel was used. (**D**) Quantitative RT-PCR analysis of *ACTBL2* expression level. cDNAs of five melanoma cell lines served as templates. The results were normalized against the results for *HPRT1* gene and A375 cells (n = 3). (**E**) The expression level of *ACTB* (ENST0000064664), *ACTG1* (ENST00000573283), and *ACTBL2* (ENST00000423391) assessed by NGS. (**F**) RNAscope analysis of five melanoma cell lines detecting mRNA coding for actbl2. Yellow arrows highlight cells with detected mRNA coding for actbl2. The results are expressed as the mean ± SD; *p* ≤ 0.05 (*).

To check if actbl2 incorporates into similar structures like β- and γ-actin, we have transfected A375 human melanoma cells with plasmids coding for β-actin, γ-actin, and actbl2 tagged at their N-terminus with 3xHA. It is noteworthy that within the coding sequences of actins, we preserved the 3′UTR regions (because this region of mRNA is important for proper localization of at least β-actin in the cell)^44–46^. All three actins were present at the leading edge of the lamellipodium and stress fibers (Fig. 5A). The DRAQ5 staining showed additional round structures within a cell body. We did not check whether they could be, e.g., lipid droplets; however, they are not *Mycoplasma* contamination as the staining pattern is different, and we routinely check the cells for this type of contamination. The high-resolution microscopy analysis revealed that HA-β actin, HA-γ actin, and HA-actbl2 were present in single actin filaments and stress fibers (Fig. 5B).

Next, we checked the expression levels of *ACTBL2*, *ACTB,* and *ACTG1* in five melanoma cell lines. Because *ACTBL2* is a gene lacking introns, we first verified that contaminating genomic DNA was not present in the RNA template. Results of one-step PCR showed that there were no qPCR products in the absence of RT-enzyme (Fig. S10), meaning that the obtained RNA was devoid of gDNA contamination. Next, PCR and qPCR analyses showed that the expression level of *ACTBL2* was relatively similar in all cell lines (Figs. 5C,D, S11). *ACTB* and *ACTG1* were expressed at the level of ca. 950 and 640 FPKM (fragments per kilobase per million mapped reads), respectively, as revealed by next gene sequencing analysis (Fig. 5E). The amount of actbl2 coding transcript equaled 1.4 FPKM in A375 cells suggesting very low *ACTBL2* expression in these cells.

Next, we detected actbl2 transcripts in the studied melanoma cell lines. Interestingly, we could detect actbl2 mRNA-positive cells in the case of A375 and Hs294T cells (Fig. 5F). However, only a subset of the cells were positive. In the case of WM1341D, SK-MEL-28, and WM9 cells, we did not observe any specific signals for actbl2 mRNA. According to the manufacturer (personal communication), the assay allows detecting mRNA for a given protein from a certain expression level. Thus, it cannot be excluded that the expression level of *ACTBL2* in these cell lines was very low across all cells. On the contrary, although the *ACTBL2* expression was also very low in the whole population of A375 and Hs294T cells, it was sufficiently high in a small subset of cells to be detected with the RNAscope procedure.

### Successful knockout of *ACTBL2* with the CRISPR/Cas9(D10A) technique and overexpression of actbl2 in A375 cells

Because we wanted to elucidate the role of actbl2 in melanoma cells, we decided to inactivate the gene coding for actbl2. We obtained three control clones (CR-CTRL) and three clones devoid of actbl2 (CR-*ACTBL2*) of A375 cells. The gDNA analysis of the obtained clones showed that several of them had changed DNA in the coding sequence for actbl2 (Fig. S12). Since there are no specific antibodies detecting actbl2^12^ (and our unpublished data), we have cloned gDNA fragments within the actbl2 coding sequence into the pAcGFP-C1 plasmid and sequenced them. This analysis revealed that five out of six alleles of CR-*ACTBL2* clones coding for actbl2 were altered in a way that ORF was shifted (Fig. S13). For the sixth allele, there was a 111-bp insertion, which did not change the ORF. However, we did not notice an altered behavior for this clone (clone no 3) relative to the rest of the CR-*ACTBL2* clones.

**Figure 6:**
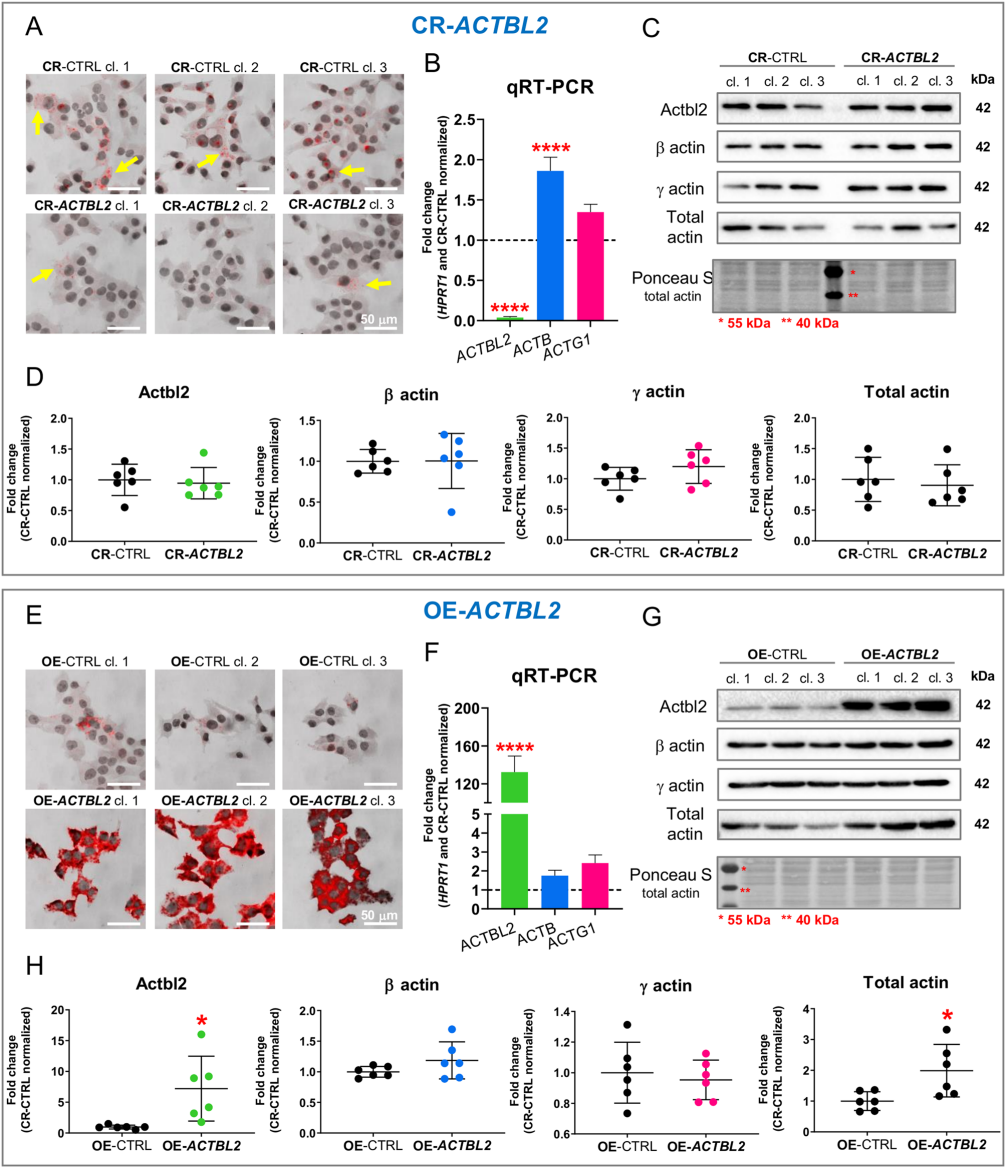
Successful knockout of *ACTBL2* and overexpression of actbl2 in A375 cells. (**A**,**E**) RNAscope analysis of CR-CTRL, CR-*ACTBL2*, OE-CTRL, and OE-*ACTBL2* clones to detect mRNA coding for actbl2. Yellow arrows highlight cells with detected mRNA coding for actbl2. (**B**,**F**) qPCR analysis of expression of *ACTBL2*, *ACTB*, and *ACTG1* in control clones and clones either devoid of actbl2 or actbl2 overexpressing (n = 3). Dotted lines represent expression levels for control cells. (**C**,**G**) Western blot analysis of lysates of CR-CTRL, CR-*ACTBL2*, OE-CTRL, and OE-*ACTBL2* cells. Membranes were incubated with antibodies directed against actbl2, β-actin, γ-actin, and total actin. Corresponding Ponceau S stainings of membranes and uncropped membranes are shown in Fig. S14. Thirty micrograms of protein was loaded in every lane. (**D**,**H**) Densitometric analyses of Western blots are presented in (**C**,**G**), respectively. Results are expressed as the mean ± SD (D,H) or mean ± SEM (B,F); *p* ≤ 0.05 (*), *p* ≤ 0.0001 (****).

Using the RNAscope assay, we evaluated the presence of cells positive for actbl2 mRNA. We observed a lower abundance of actbl2 mRNA-positive cells for CR-*ACTBL2* clones than control cells (Fig. 6A). Similarly, the qPCR analysis also showed a drastically decreased *ACTBL2* level in CR-*ACTBL2* clones compared to control cells (Fig. 6B). Interestingly, *ACTB* but not *ACTG1* level was significantly increased when compared to CR-CTRL clones. Finally, by Western blotting, we checked the amounts of actbl2, β- and γ-actin, and the total level of actin. As stated earlier, because actbl2-recognizing antibodies are not specific, there was a band for CR-*ACTBL2* clones (Figs. 6C and S14). There were no differences between control and cells devoid of actbl2 in β-actin, γ-actin, and total actin levels (Fig. 6C,D).

Due to the low expression level of *ACTBL2* in melanoma cells, we thought that *ACTBL2* knockout might not give precise answers to questions about the role of actbl2 in the motility of melanoma cells. Thus, we obtained three control clones (OE-CTRL) and three clones overexpressing actbl2 (OE-*ACTBL2*) by stable transfection of A375 cells. For this purpose, we used pLVX-hPGK-Puro—an “empty” plasmid—and pLVX-hPGK-Puro-actbl2 3′UTR plasmids. We used the hPGK promoter as it is a moderate promoter compared to the CMV promoter^47^, which is an advantage when considering the efficient, stable expression of proteins. Again, we preserved the 3′UTR region of the sequence coding for actbl2. We used untagged actbl2 construct, because the main differences between actin isoforms are within the N-terminus, which is mirrored at least partially in diverse regulation of isoactins^5,12^, resulting in changed functionality. We did not want to influence the potential functionality of actbl2 by introducing a tag at its N-terminus. Also, we did not tag actbl2 at the C-terminus, as it was shown that this might lead to perturbations in actin folding^48^.

RNAscope assay done on OE-CTRL and OE-*ACTBL2* clones showed a very high level for actbl2 mRNA in the OE-*ACTBL2* cells (Fig. 6E), which was corroborated by qPCR analysis (Fig. 6F). However, there were no alterations in the *ACTB* and *ACTG1* expression levels in OE-*ACTBL2* cells relative to control cells. Interestingly, antibodies directed against actbl2 recognized the overexpressed actin (Figs. 6G and S14). Based on the results presented in Fig. 6C,G, we conclude that these antibodies recognize not only actbl2 but also other actin isoforms. The β- and γ-actin levels remained unchanged, whereas the total actin level was increased (Figs. 6G and S14). The densitometric analysis of the membranes confirmed these results. Significantly higher levels were noted for actbl2 and total actin for OE-*ACTBL2* cells compared to control cells (Fig. 6G,H). These results show that we obtained stable clones with *ACTBL2* knockout and overexpression of actbl2. Moreover, unlike CR-*ACTBL2* cells, OE-*ACTBL2* cells had an altered total actin level.

### *ACTBL2* knockout, and overexpression of actbl2 differently affect 2D migration abilities of A375 cells

**Figure 7:**
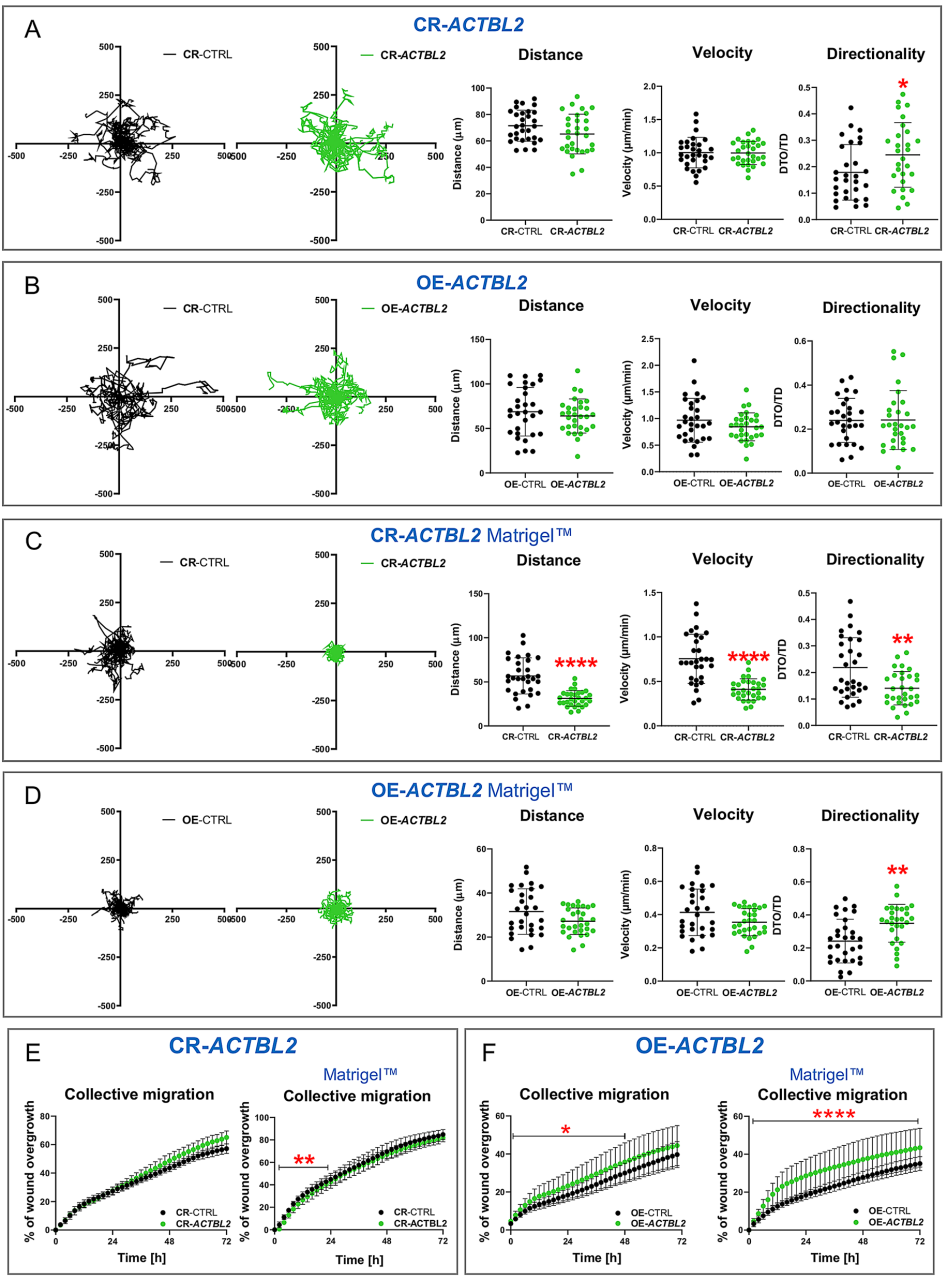
The lack of actbl2 and actbl2 overexpression differently affects the 2D migration of A375 cells. (**A**,**B**) Spontaneous 2D migration of control cells and cells either devoid of actbl2 or overexpressing actbl2. The cells were seeded onto IncuCyte ImageLock plates. The migration assay was performed using IncuCyte Live Cell Analysis Imaging System for 72 h with images taken every 2 h. Based on single trajectories of the cells, distance, velocity, and directionality were measured (n = 30). (**C**,**D**) As presented in A and B, an analogous analysis was done for the same clones seeded into plates coated with Matrigel (n = 30). (**E**,**F**) The control clones and CR-*ACTBL2* and OE-*ACTBL2* clones were subjected to collective migration assay for 72 h (n = 3). The cells were seeded on non-coated or Matrigel-coated substratum. Representative photomicrographs are presented in Fig. S15. Results are expressed as the mean ± SD; *p* ≤ 0.05 (*), *p* ≤ 0.01 (**), *p* ≤ 0.0001 (****).

Actins are critical players in cell motility, so we tested the 2D motility of the analyzed cells. Spontaneous migration assays were conducted over 72 h on CR-CTRL and CR-*ACTBL2* cells. Several parameters characterizing the cell movement have been calculated. Distance means the total path traveled by single-cell within 72 h. The velocity is the displacement of the cell divided by the time interval. Directionality (tortuosity) is a ratio between straight-line displacement between start and endpoints of movement and the total path traveled by single-cell. Plotted single-cell trajectories did not show any differences between the two types of clones. However, the directionality of CR-*ACTBL2* cells was increased when compared to control cells (Fig. 7A). Interestingly, the velocities of CR-CTRL and CR-*ACTBL2* cells were similar. In the case of overexpression of actbl2, we did not notice any alterations for OE-*ACTBL2* cells in terms of covered distances, velocity, or directionality compared to OE-CTRL clones (Fig. 7B).

Because melanoma cells in initial phases of tumor transformation reside on the basement membrane and, during tumorigenesis progression, have to transverse it, we also checked their 2D migration on a surface coated with Matrigel, which resembles the extracellular basement membrane’s matrix. CR-*ACTBL2* cells’ covered distances, velocity, and directionality were profoundly affected compared to control cells (Fig. 7C). On the other hand, OE-*ACTBL2* clones had improved directionality, but the distances covered by them and their velocity remained unchanged compared to control clones (Fig. 7D).

Finally, we checked the collective migration of studied cells. In the case of the cells seeded on a non-coated surface, we did not notice any differences in the scratch closure rate between control cells and clones devoid of actbl2 (Figs. 7E and S15A,B). We noted a significant difference only for the first 24 h of wound healing assay in the case of CR-*ACTBL2* cells seeded on Matrigel-coated surface, which closed the scratch slower than control cells. This might be connected with impaired directionality for these cells. For OE-*ACTBL2* cells, we noted a faster scratch closure over the first 48 h on a non-coated surface compared to control clones (Figs. 7F and S15C). However, OE-*ACTBL2* cells closed the scratch faster than control cells over 72 h when the cells were seeded on a Matrigel-coated surface (Figs. 7F and S15D). Altogether these results imply that the lack of actbl2 had more severe consequences in terms of spontaneous 2D migration than overexpression of actbl2. However, the cells overexpressing actbl2 migrated collectively better than control clones.

### Invasion potential is lowered in CR-*ACTBL2* cells, and the formation of protrusive structures is changed in these cells

**Figure 8:**
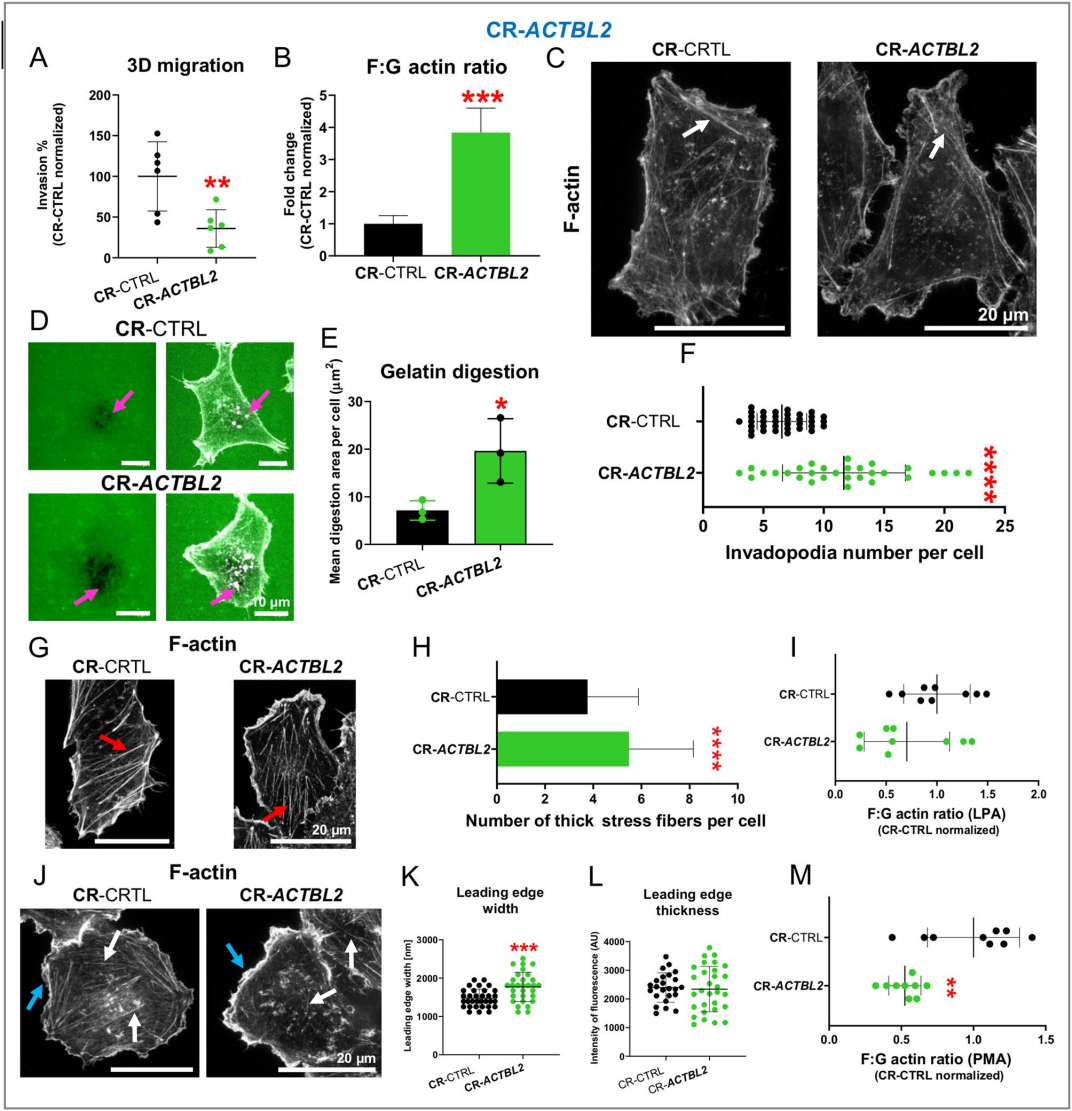
Invasion potential of CR-*ACTBL2* clones was diminished, accompanied by alterations in actin cytoskeleton architecture upon LPA and PMA treatment. (**A**) Invasion/3D migration potential was measured for CR-CTRL and CR-*ACTBL2* cells (n = 6). (**B**) 24 h after seeding, the cells were lysed, and the F:G actin ratio was estimated (n = 8–9). (**C**) STED microscopic analysis of CR-CTRL and CR-*ACTBL2* cells. The cells were fixed and stained with phalloidin-Abberior Star Red. White arrows highlight stress fibers. (**D**) The cells were seeded on fluorescently labeled gelatin. 12 h later, the cells were fixed and stained with phalloidin Alexa Fluor 568. Pink arrows point at invadopodia and the digested area. The digested area (**E**) and the number of invadopodia (**F**) were calculated based on captured images (n = 30). (**G**) After 24 h of starvation, the studied clones were treated for 10 min with 1 μM LPA. Next, they were fixed, stained with phalloidin-Abberior Star Red, and subjected to STED microscopy. Red arrows point at thick actin bundles/stress fibers. (**H**) Thick microfilament bundles were calculated from images taken using a confocal microscope (n = 60). Representative photos are shown in Fig. S16B. (**I**) Upon LPA treatment, the cells were lysed and subjected to F:G actin ratio assay (n = 9). (**J**) 24 h upon starvation, the cells were incubated with 100 nM PMA for 5 min. Next, they were fixed and stained with phalloidin-Abberior Star Red. The following photos were taken with STED microscopy. Blue arrows highlight lamellipodia, and white arrows indicate stress fibers. The leading-edge width (**K**) and thickness (**L**) were calculated based on confocal microscopy images (n = 30). Representative photos are shown in Fig. S16C. (**M**) The F:G actin ratio was measured for CR-CTRL and CR-*ACTBL2* cells after the addition of 100 nM PMA for 5 min (n = 9). Results are expressed as the mean ± SD; *p* ≤ 0.05 (*), *p* ≤ 0.01 (**), *p* ≤ 0.001 (***), *p* ≤ 0.0001 (****).

After checking 2D migration, we looked at the 3D migration/invasion potential of cells devoid of *ACTBL2*. We observed that the CR-*ACTBL2* cells had diminished invasion potential compared to control cells (Fig. 8A). Because actin polymerization status decides about cells’ motility^49,50^, we estimated the F:G actin ratio, which was much higher for CR-*ACTBL2* cells than in control cells (Fig. 8B). The microscopic analysis with the help of STED and confocal microscopy of the cells stained with fluorescently-labeled phalloidin showed that the CR-*ACTBL2* cells had a more prominent transverse arcs network than the control cells (Figs. 8C and S16A).

Next, we looked at invadopodia as these structures are crucial for the invasion of cells. Gelatin-FITC digestion assay was performed to evaluate invadopodia formation in the cells. The cells growing on fluorescently labelled gelatin were fixed and stained to detect F-actin. Next, the photos were taken with the confocal microscope. We observed that the digested area in gelatin-FITC was more prominent for the cells devoid of actbl2, and they formed a higher number of invadopodia than control clones (Fig. 8D–F). To force the cells to form stress fibers, we stimulated them with lysophosphatidic acid (LPA)^51^. The CR-*ACTBL2* cells formed a higher number of thick actin bundles than control cells (Figs. 8G,H and S16B). The F:G actin ratio was, however, unchanged in these cells (Fig. 8I). Eventually, we treated the cells to stimulate the formation of lamellipodia^52^. We observed a more pronounced leading edge in the cells lacking actbl2 (Figs. 8J and S16C).

Interestingly, in both STED and confocal analyses, we found that there was a subtle microfilament network in control cells, which was not as prominent as in the cells devoid of actbl2. The quantitative analysis of the leading edge width (Fig. 8K) and thickness (Fig. 8L) showed that the former was increased, but the latter was unchanged for CR-*ACTBL2* cells when compared to CR-CTRL clones (Fig. 8L). Finally, the F:G actin ratio revealed that CR-*ACTBL2* cells had a lower ratio than control clones (Fig. 8M). These observations show that the lack of *ACTBL2* impairs the invasion abilities of the cells, although these cells produce more invadopodia. Moreover, CR-*ACTBL2* cells are characterized by the changed formation of actin-based protrusive structures.

### Overexpression of actbl2 enhances invasion of A375 cells and have partly reverse effects on the formation of protrusive structures in comparison to *ACTBL2* knockout

**Figure 9:**
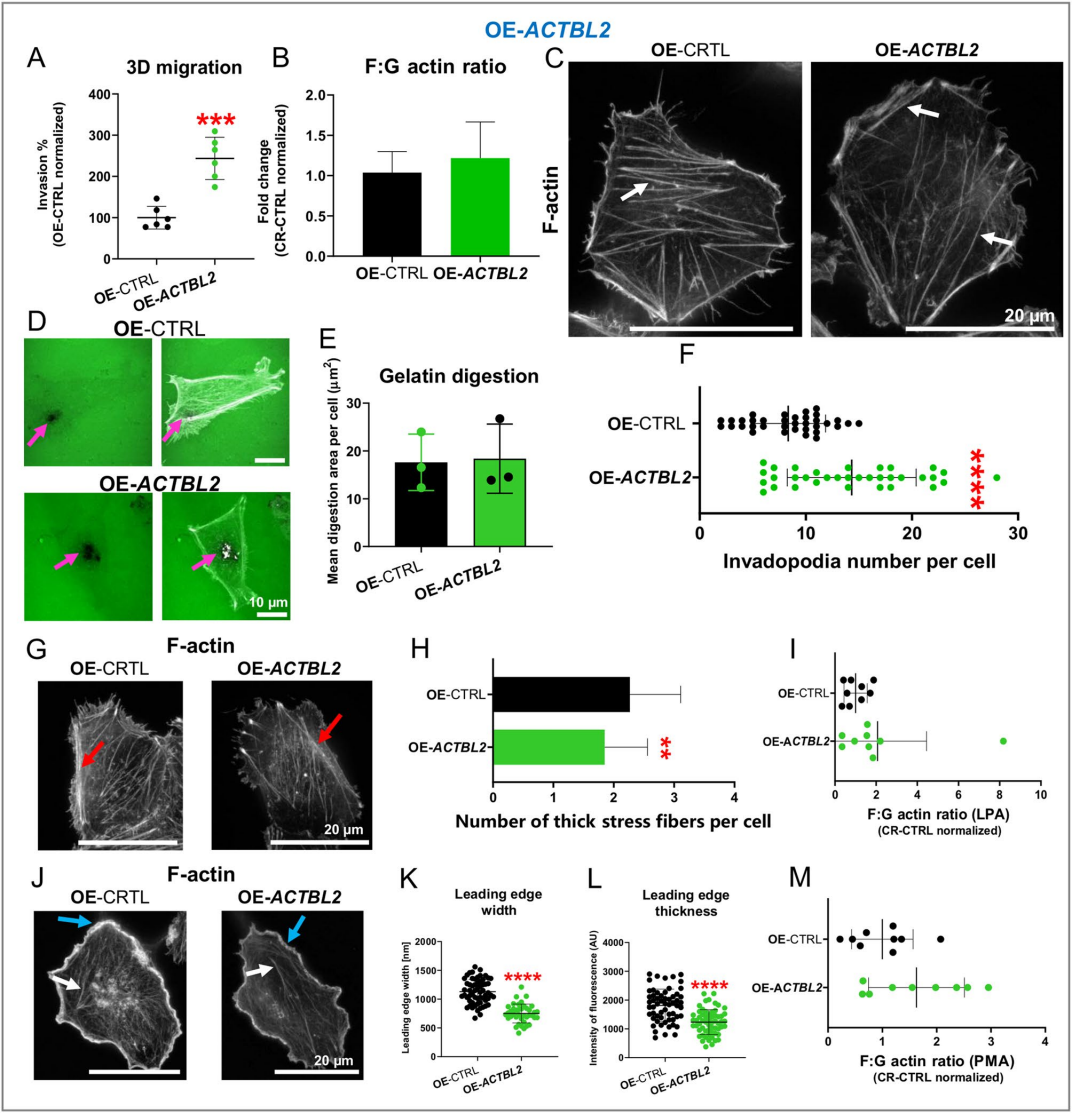
Overexpression of actbl2 leads to improved invasion abilities and reverse effects on actin cytoskeleton architecture in comparison to *ACTBL2* knockout. (**A**) The invasion potential of studied clones was estimated (n = 6). (**B**) F:G actin ratio was evaluated for OE-CTRL and OE-*ACBTL2* cells 24 h upon seeding the cells (n = 9). (**C**) The cells fixed 24 h after seeding them on coverslips were stained with phalloidin-Abberior Star Red and subjected to STED microscopy analysis. White arrows highlight stress fibers. (**D**) The cells were seeded on fluorescently labeled gelatin, and 12 h later, they were fixed and stained with phalloidin Alexa Fluor 568. Pink arrows point at invadopodia and the digested area. The area of digested FITC-gelatin (**E**) and the invadopodia number (**F**) formed by OE-CTRL and OE-*ACTBL2* cells were measured (n = 30). (**G**) After starvation for 24 h, the cells were treated with 1 μM LPA for 10 min, fixed, stained with phalloidin-Abberior Star Red, and analyzed using a STED microscope. Red arrows highlight thick actin bundles. (**H**) The number of thick actin bundles formed by OE-CTRL and OE-*ACBTL2* upon LPA treatment (n = 60). Representative photos are shown in Fig. S16E. (**I**) The F:G actin ratio was evaluated for cells exposed to LPA (n = 9). (**J**) Upon incubating the studied clones with 100 nM PMA for 5 min, the cells were fixed and stained with phalloidin-Abberior Star Red. The cells were then analyzed by STED microscopy. Blue arrows point at lamellipodia and white arrows at stress fibers. The leading-edge width (**K**) and thickness (**L**) were calculated using confocal microscopy images (n = 30). Representative photos are shown in Fig. S16F. (**M**) The F:G actin ratio was measured for OE-CTRL and OE-*ACTBL2* cells upon PMA stimulation (n = 9). Results are expressed as the mean ± SD; *p* ≤ 0.01 (**), *p* ≤ 0.001 (***), *p* ≤ 0.0001 (****).

In the next step, we evaluated the impact of actbl2 overexpression on cell invasion abilities. OE-*ACTBL2* cells had an increased invasion potential over control cells (Fig. 9A). The F:G actin ratio of OE-*ACTBL2* cells was unchanged when compared to OE-CTRL clones (Fig. 9B). STED and confocal analyses showed less abundant stress fibers formation in the cells overexpressing actbl2 (Figs. 9C and S16D). Subsequently, we performed gelatin-FITC digestion assay to evalute invadopodia formation (Fig. 9D). The OE-*ACTBL2* cells’ digestion area was unaltered (Fig. 9E), and the number of formed invadopodia was surprisingly increased in comparison to OE-CTRL cells (Fig. 9F).

Next, we looked at the actin organization in the studied cells upon LPA stimulation. The addition of this reagent for 10 min at the concentration of 1 μM led to less manifested stress fibers in OE-*ACTBL2* cells, as stated with the help of STED and confocal microscopy (Figs. 9G and S16E). The cells overexpressing acbtl2 had indeed less thick actin bundles than control cells (Fig. 9H). However, the F:G actin ratio remained unchanged in these clones compared to control cells (Fig. 9I). We also treated the cells to challenge the formation of lamellipodia. Microscopic analysis revealed less prominent leading edges in OE-*ACTBL2* cells when compared to control cells (Figs. 9J and S16F). The study of leading-edge width and thickness confirmed this observation (Fig. 9K,L). Both parameters were lowered in the cells overexpressing actbl2 in comparison to control cells. Finally, we checked the F:G actin ratio. We found that there were no differences between OE-*ACTBL2* and control cells (Fig. 9M). In conclusion, these data show that actbl2 overexpression increased A375 cells’ invasion and affected the formation of protrusive structures built of F-actin. Nevertheless, this effect was mainly the reverse to those observed for cells devoid of actbl2.

### Knockout of *ACTBL2* led to the pronounced formation of focal adhesions in A375 cells under control conditions and partly upon LPA stimulation but not in response to PMA addition

In one of our previous experiments, we observed that the cells lacking β- or γ-actin had changed the formation of focal adhesions (FAs)^8^. That is why we evaluated the impact of *ACTBL2* knockout on FAs formation. We tested cells grown in the presence and the absence of FBS. The cells were fixed and stained with antibodies recognizing either α-Parvin or VASP. α-Parvin is a constituent of focal adhesion^53^, and VASP is an actin nucleator also present in FAs^8,49^. α-Parvin in A375 cells is present solely in FAs. In contrast, VASP is present not only in FAs but is also found submembranous together with actin aggregates, which we consider as nascent FAs, and in the cell body^8^.

**Figure 10:**
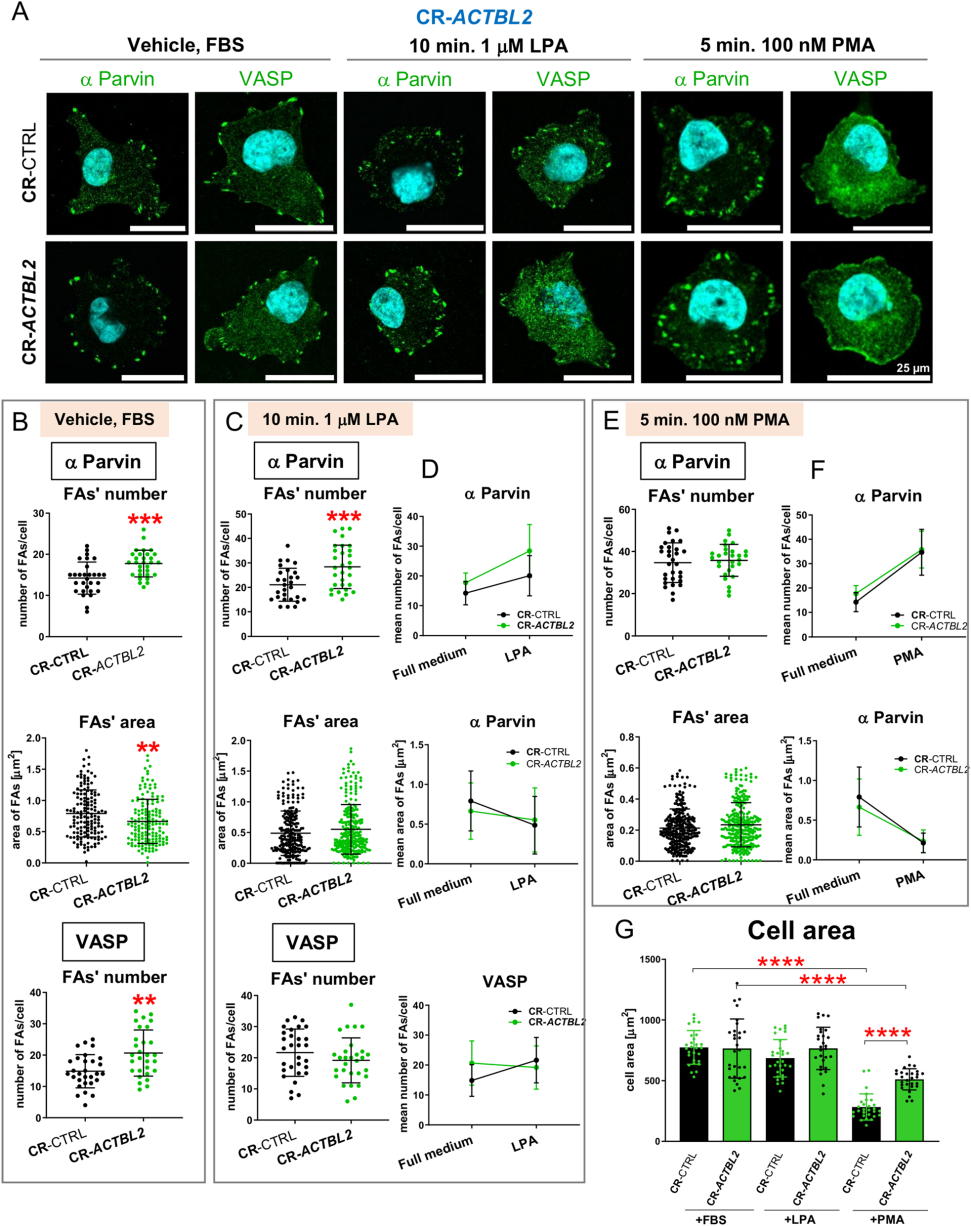
The formation of focal adhesions is changed in CR-*ACTBL2* cells relative to CR-CTRL clones. (**A**) The cells were seeded on coverslips. Some of them were treated with either 1 μM LPA for 10 min or 100 nM PMA for 5 min. 24 h later, they were fixed. The cells were stained with antibodies recognizing either α-Parvin or VASP. (**B**,**C**,**E**) Based on captured photos, the number of FAs and their area were estimated (n = 30). (**D**,**F**) Slope graphs presenting trends upon LPA or PMA stimulation in the number and surface area of FAs. (**G**) The cell area surface of the cells grown in the presence of FBS or treated with either LPA or PMA was measured (n = 30). Results are expressed as the mean ± SD; *p* ≤ 0.01 (**), *p* ≤ 0.001 (***), *p* ≤ 0.0001 (****).

We calculated the number of FAs and their surface areas (Fig. 10A). We found that the number of α-Parvin-rich FAs was increased, but their surface area was decreased in CR-*ACTBL2* cells compared to control cells (Fig. 10B). The number of VASP-rich FAs was also elevated for CR-*ACTBL2* cells when compared to CR-CTRL clones. We also evaluated the same parameters for the cells incubated for 24 h in a starvation medium (Fig. S17). Differently to full medium conditions, we observed that the number of α-Parvin-rich FAs and their surface area was unchanged compared to control cells (Fig. S17B). For VASP-rich FAs, we found no changes as compared to control cells (Fig. S17B).

Upon LPA stimulation, the number of α-Parvin-rich FAs was higher in the cells devoid of actbl2 than in control clones (Fig. 10C). However, the surface area of these structures and the number of VASP-rich FAs in these cells were unchanged compared to control cells (Fig. 10C). The slope graphs show that FAs formation by CR-*ACTBL2* cells upon stimulation with LPA increased the number of α-Parvin-rich FAs, but their surface area was diminished, just like in the case of control cells (Fig. 10D). However, different to the control cells, CR-*ACTBL2* cells tended to form VASP-rich FAs, i.e., while the control cells formed more of these structures upon LPA stimulation, the cells devoid of actbl2 exhibited a similar number of VASP-rich FAs under both conditions. Again, an analogous analysis was done for the cells kept without FBS versus the cells treated with LPA, which gave different outcomes (Fig. S17C), because the results for +FBS and -FBS conditions were diverse (Figs. 10C and S17B).

The treatment of the cells with PMA resulted in no changes in the number of α-Parvin-rich FAs and their surface area for CR-ACTBL2 cells compared to control clones (Fig. 10E). PMA stimulation led to the redistribution of VASP in the cells, making it impossible to quantify the VASP-rich FAs (Fig. 10A). Eventually, we plotted slope graphs presenting the tendencies in α-Parvin-rich FAs formation. They showed that both the number of VASP-rich FAs and their area changed after the addition of PMA in the same way for control cells and cells devoid of actbl2 (Fig. 10F). The same observation was done based on the slope graphs comparing the results obtained for the cells stimulated with PMA and grown for 24 h without FBS (Fig. S17D).

Finally, we looked at the cell surface area as the number of FAs determines the spreading of a cell^13^. The smallest surface area was observed for the cells treated with PMA compared to control cells (Fig. 10G). The surface area of the cells was not changed upon LPA addition and we could detect the differences between the control and CR-*ACTBL2* cells only in the case of the treatment with PMA. For the cells kept for 24 h without FBS, we noted similar results for cells grown in full medium (Fig. S17E). Data presented here imply that the lack of actbl2 influences FAs formation.

### Overexpression of actbl2 changes FAs formation rate in a reverse way than in the case of ACTBL2 knockout

**Figure 11:**
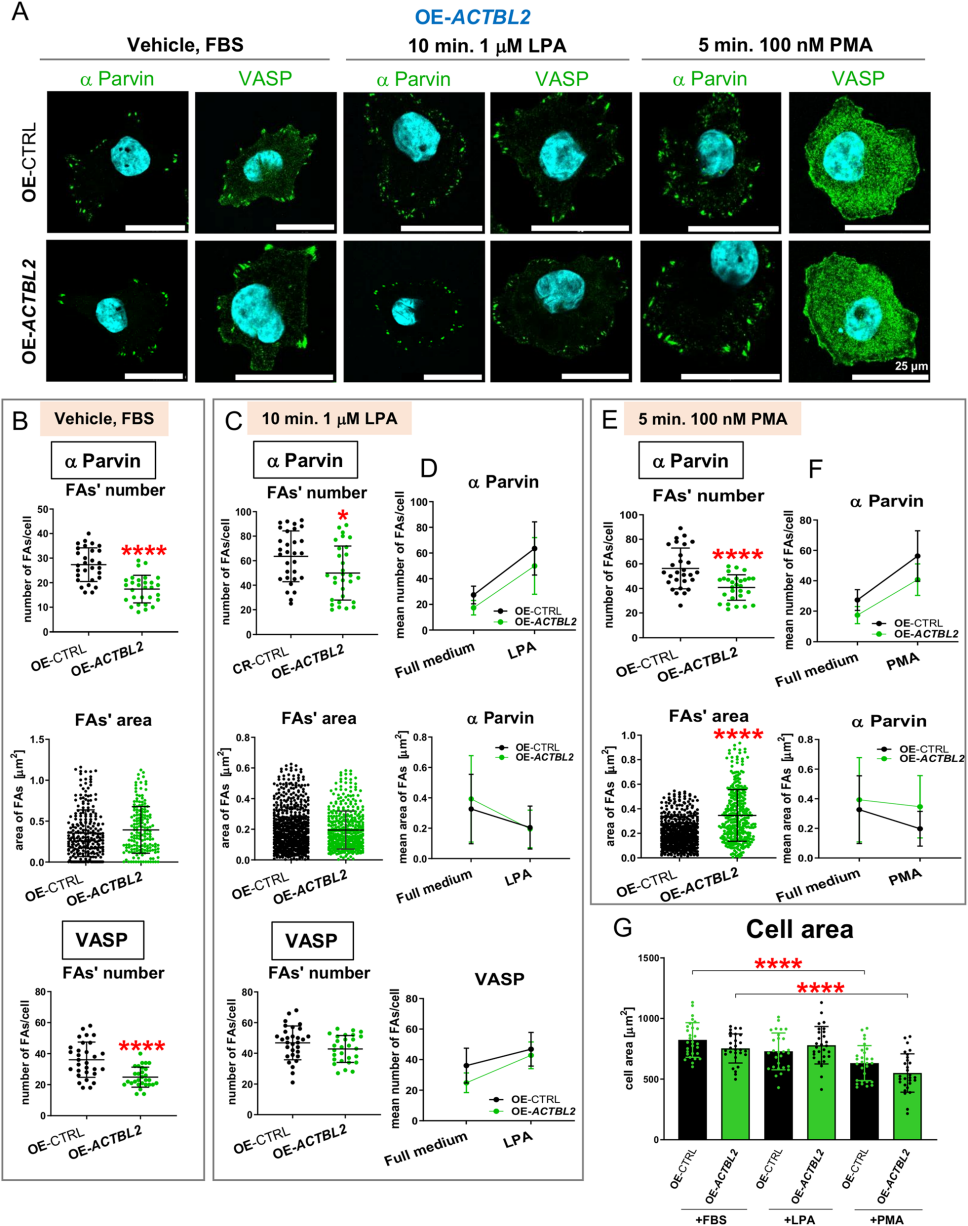
Overexpression of actbl2 resulted in reverse effects on FAs formation relative to *ACTBL2* knockout cells. (**A**) Upon seeding the cells on the coverslips and treatment, some of them with either 1 μM LPA for 10 min or 100 nM PMA for 5 min, the cells were fixed and stained with antibodies directed against either α-Parvin or VASP. (**B**,**C**,**E**) After taking the photos of stained cells presented in A, the FAs number and their area were measured and presented as bar charts (n = 30). (**D**,**F**) Slope graphs showing trends upon LPA or PMA stimulation in the number and surface area of focal adhesion. (**G**) The cell surface area of OE-CTRL and OE-*ACTBL2* cells growing in the presence of FBS or upon treatment with either LPA or PMA was measured (n = 30). Results are expressed as the mean ± SD; *p* ≤ 0.05 (*), *p* ≤ 0.0001 (****).

Next, we evaluated FAs formation in the cells overexpressing actbl2. Like in the case of CR-clones, we took microphotographs of the cells using a confocal microscope (Fig. 11A). The cells were stained with antibodies directed against either α-Parvin or VASP. For OE-*ACTBL2*, we observed a lowered number of α-Parvin-rich FAs compared to control cells (Fig. 11B). However, their surface area was unchanged for the cells overexpressing actbl2. The decreased number of VASP-rich FAs was also noted for OE-*ACTBL2* clones compared to control cells (Fig. 11B). Moreover, we evaluated the same parameters in the cells incubated for 24 h in the starvation medium. Similar to the cells kept in full medium, we observed that the number of α-Parvin-rich FAs was significantly diminished in comparison to control cells. At the same time, their surface area was unchanged (Fig. S18A,B). In the case of VASP-rich FAs, their number was also lowered for OE-*ACTBL2* cells compared to control clones (Fig. S18A,B)

Upon LPA addition, the number of α-Parvin-rich FAs dropped in the case of actbl2 overexpressing cells, but their surface area remained unchanged (Fig. 11C). The number of VASP-rich FAs was also unaltered in OE-*ACTBL2* clones compared to control clones (Fig. 11C). The slope graphs showed that upon LPA treatment, OE-*ACTBL2* cells behaved similarly to control cells (Fig. 11D). Similar conclusions were drawn when the results for the cells kept without FBS were compared with the outcomes obtained for the cells stimulated with LPA (Fig. S18C).

Then, we examined the number of α-Parvin-rich FAs after PMA administration. Their number was diminished for OE-*ACTBL2* clones, but their surface area was increased (Fig. 11E). The slope graphs showed that actbl2 overexpressing cells behaved similarly to the control cells in terms of α-Parvin-rich FAs formation (Fig. 11F). The same observation was done based on the slope graphs comparing the results obtained for the cells stimulated with PMA and grown for 24 h without FBS (Fig. S18D).

Finally, we evaluated the cell surface area of the cells subjected to the conditions described here. Only in PMA stimulation, we observed differences in the control conditions (Fig. 11G). Both control and OE-*ACTBL2* cells were smaller when compared to the cells grown in the presence of FBS. There were no differences between OE-CTRL and OE-*ACTBL2* for all the tested conditions. In the case of the cells kept for 24 h without FBS, surprisingly, we noted that the surface area of OE-*ACTBL2* cells was significantly increased upon LPA stimulation in comparison to non-stimulated cells (Fig. S18E). The results show that actbl2 overexpression changed the number and size of formed FAs; however, the effect of this genetic manipulation was generally reverse to the *ACTBL2* knockout.

## Discussion

This study was performed to understand the role of the understudied, newly discovered actin isoform actbl2 in melanoma cells. There is an abundance of data regarding the other six actin isoforms concerning their function, both in normal and tumor cells, but the origin and role of β actin-like protein 2 has, until now, been a mystery. Because so far there has only been one study on actbl2 functionality^14^, this research contributes to the understanding of evolutionary history, function, and influence of actbl2 on tumorigenesis, starting with its role in melanoma cells.

Phylogenetic analyses based on amino acid sequences showed that two actin classes, non-muscle (cytoplasmic) and muscle^54^, are separate lineages, which could evolve independently in vertebrates from invertebrate groups. In contrast to that, the trees based on the nucleotide sequence alignment grouped these classes. Still, this might be associated with a phylogenetic artifact due to a higher evolutionary rate of nucleotide sequences than amino acid sequences. The separation of these classes before the vertebrate origin is in agreement with previous studies^55–58^. However, in contrast to previous analyses^59,60^ and in agreement with newer one^61^, our results indicate that not only the duplication of ancestral muscle actin into striated and smooth muscle actin but also the emergence of all canonical actin groups could occur in early vertebrate evolution before the separation of cartilaginous fishes (chondrichthyans) and Teleostomi (included bony fishes and tetrapods) or the divergence of at least bony fishes and tetrapods (in the case of *ACTA2* group).

Interestingly, the grouping of tunicate sequences with those of *ACTA1* suggests that some actin lineages could diverge even earlier. The rapid evolution of actins in the vertebrate origin was most likely associated with the whole genome duplication, whose two rounds occurred in early vertebrate evolution^62–64^. The occurrence of new gene copies led to their sub- or neo-functionalization. Thanks to that, they acquired new roles or specializations in the cell.

The inclusion of many more sequences and various approaches helped us to better resolve the actin phylogeny. Phylogenetic relationships inferred between actin groups turned out to follow their functional characteristics. Not only non-muscle (cytoplasmic) and muscle actins were separated but also striated muscle actins (*ACTA1* + *ACTC1*) and smooth muscle actins (*ACTA2* + *ACTG2*) created distinct and supported clades. Recent synteny analyses showed that within the already established actin groups, additional groups could be recognized^61^. The two new groups named ε1 (*ACTE1*) and α4 (*ACTA4*) were grouped in our phylogenies with γ smooth muscle actins (*ACTG2*) and striated muscle actins (*ACTA1* or *ACTC1*), respectively.

Sequences similar to human *ACTBL2* created a separate clade related to the β non-muscle group (*ACTB*) and evolved in early vertebrates, likely before the divergence of cartilaginous fishes and the lineage of bony fishes and tetrapods. *ACTBL2* genes, especially those in mammals and other amniotes (birds and reptiles), were likely retrotransposed and did not contain introns, as indicated in NCBI Gene and Ensemble databases. Thus, the probable scenario is that after duplication of actin genes before the radiation of fishes, one gene set evolved into the *ACTB* lineage and the other into the *ACTBL2* lineage. Intron-containing genes showing similarity to *ACTBL2* were preserved in fishes, but in amniotes, the genes began to lose introns via retroposition. It cannot be ruled out that the retroposition of actins occurred several times in different taxonomic groups. The sequences of *ACTBL2* and *ACTB* were also subjected to the highest substitution rate, 2–8 times greater than other actin groups.

In contrast, the muscle actins evolved with the slowest and the most homogenous rate. Among all actins, *ACTBL2* revealed the most extensive number of substitutions changing amino acid physicochemical properties, which can be linked with the evolution of their unique functions and different roles in the cell. These specific properties were revealed in our experimental studies.

The results obtained here showing incorporation of HA-actbl2 into the same structures as in the case of HA-β actin and HA-γ actin are the same as those published recently, in which we showed the presence of β- and γ-actin in stress fibers and lamellipodia of non-manipulated melanoma cells^8^. Multiple groups have reported similar results, e.g., Dugina and colleagues showed the presence of β-actin in fibroblasts at cell to cell contact sites and in stress fibers, whereas γ-actin was reported in the lamellipodium^65^. We cannot exclude the possibility that the similar localization of actbl2 to β-actin and γ-actin is caused by the tagging of the N-terminus, within which there are the major differences between isoactins concerning amino acid sequence^5,12^, what could influence localization and function of isoactin. However, our main goal here was to check if, under the same conditions, there will be any differences in localization of actbl2 in comparison to β-actin and γ-actin.

Genetically manipulating cells, e.g., by gene expression silencing, overexpression, or edition, has been used in numerous studies focused on the roles of β- and γ-actin in cells^65–69^. We also applied these well-established methods in the study of the actbl2 function. Upon initial selection, by checking the mRNA levels of actbl2 in different melanoma cell lines, we induced knockout and overexpression of this isoform in A375 cells, which we confirmed on the mRNA level. Checking the protein levels proved to be a challenge, as there is no specific antibody to this actin. The antibody directed to actbl2 also recognizes other actins, but there was an increase in total actin only in the case of OE-*ACTBL2* clones, whereas a decrease was not shown in the CR-*ACTBL2* clones. It has been shown that upon silencing of one actin isoform, there is a compensation in the level of another isoactin^8,68,70–72^. We observed a similar phenomenon upon the silencing of actbl2 when the transcript level of *ACTB* but not *ACTG* was significantly elevated. However, there was no increase in the β-actin protein level.

The much increased level of total actin is an exciting observation suggesting differential, when compared to the other two non-muscle actins, regulation of the maintenance of the total actin level in the case of *ACTBL2* expression. On the one hand, it was noted many times that lowering the expression of one non-muscle actin isoform resulted in overexpression of other isoforms to restore the level of total actin^8,68,70–72^. On the other hand, we failed to achieve stable overexpression of β- or γ-actin in different tumor cell lines^73,74^ (and our unpublished data) using standard procedures applied here as well. Lately, Garner et al.^68^ showed that exchanging the sequence coding for unlabeled β-actin with the sequence encoding GFP-β-actin in the *ACTB* locus led to a statistically significant increase in the γ actin level. This was explained by the compensation of loss of unlabeled β-actin restoring the full functionality of the actin apparatus, at least in terms of the migratory abilities of HL-60 cells. Altogether, it implies that the actbl2 expression might not be so strictly controlled as in the case of other actins^5^, and its functioning is not entirely overlapping with other actins.

Interestingly, RNAscope analysis showed a subset of A375 and Hs294T cells exhibiting a high actbl2 mRNA level. No similar research was done so far on tumor cells regarding β- or γ-actin mRNA distribution in the whole population of a given cell line. Intriguingly, among the cell lines tested here, A375 and Hs294T had the highest migration potential^75^. We show here that actbl2 presence in melanoma cells is meager in comparison to β and γ actin. Taking into account that actbl2 evolves at the highest and most heterogeneous rate among all isoactins present in humans, which is presented in this study, the very low abundance of actbl2 can be interpreted from an evolutionary point of view. The presence of highly-expressed actins evolving at slower rates can allow for faster evolution of lowly-expressed actins to increase the adaptational potential of organisms^76^. Though we show here that *ACTBL2* is expressed at a very low level, several studies prove the existence of actbl2 at the protein level (Table S3, Fig. S9). However, in these studies, the level of actbl2 was not analyzed. Some of them report that actbl2 is subjected to the same posttranslational modifications, e.g., ubiquitination or phosphorylation, like the other isoactins are^5^.

Migration is a complex process regulated by different forces, and it has been shown that upon manipulation of the level of actin, the migration abilities of the cells can change^8,72^. With actbl2, Hoedebeck et al.^14^ have shown that upon silencing of actbl2, the migration abilities of the cells were impeded. We saw similarly impaired 2D migration in CR-*ACTBL2* clones, but only when cells were cultured on Matrigel (not on plastic)^8^. Previously, we reported a drop in a distance traversed by cells on plastic only in the case of CR-*ACTG1* clones (A375 cells with *ACTG1* knockout), but not in CR-*ACTB* clones (A375 cells with *ACTB* knockout), suggesting a similar behavior of CR-*ACTBL2* to CR-*ACTG1* cells ^8^. We did not see opposite results for the OE-*ACTBL2* clones, with unchanged 2D migration on Matrigel in terms of distance. Still, we did see an increase in directionality on Matrigel upon overexpression of actbl2. These results indicate that the loss of actbl2 expression had a more severe impact on cell migration than induction of overexpression. When performing a collective migration assay, where cells interact with one another and present different force types than in spontaneous movement, we saw a statistically significant increase in the wound healing by the OE-*ACTBL2* clones when compared to control, which points well to the fact that the interactions in the collective migration differ significantly when compared to 2D migration. In the case of CR-*ACTBL2*, we did not notice any effect on collective migration and the results differ in this matter when compared to CR-*ACTB* cells, which closed the scratch on plastic faster than control cells^8^.

The ability of the cells to migrate in 3D is another vital trait of tumor cells. It has been shown for the other two non-muscle actins, that upon silencing of their expression, the cells’ invasion abilities are decreased, suggesting the co-operation effect of both actins in the process of invasion^8^. Dugina and colleagues showed that lack of β-actin, but not γ-actin, also caused impairment in invasion abilities^67^. These discrepancies could be due to the different models used in the studies, resulting in differing ABPs regulation. In our research, the knockout of actbl2 resulted in impaired invasion abilities of CR-*ACTBL2* clones.

In contrast, in OE-*ACTBL2* cells, we observed increased invasive potential. Surprisingly, both sets of clones had a significantly increased number of invadopodia, suggesting that upon silencing, there could be a differential regulation of invasive structures driven by different signaling pathways^77^. We observed a similar positive correlation between impaired invasion and high invadopodia number for cells devoid of β- or γ-actin^8^. The activity of metalloproteinases seems to be increased in the CR-*ACTBL2* cells, with enhanced gelatin digestion properties, whereas in OE-*ACTBL2* cells, we did not see changes compared to controls. This again points to the more significant changes happening in the cell upon knockout of *ACTBL2*.

Here we show that upon stimulation with LPA, the number of thick stress fibers in the CR-*ACTBL2* increases, and the opposite effects were seen in OE-*ACTBL2* cells. Surprisingly, there was no difference in the F:G ratio seen in both cases. Similar results upon LPA treatment were noted for cells without β- or γ-actin^8^. Upon stimulation with PMA, the leading edge width was significantly increased for CR-*ACTBL2* cells, and we also saw a significant decrease in the F:G ratio, which could mean that in the absence of actbl2, there is a lesser actin turnover. For the OE-*ACTBL2* clones, we saw opposite effects, as the leading edge width and thickness were decreased when compared to control cells, where the F:G actin ratio was not significantly changed, but with a tendency towards higher F:G ratio. Earlier, we showed that CR-*ACTG1* but not CR-*ACTB* cells had narrower and thinner leading edges^8^.

We also checked the formation of focal adhesions in the cell, as their structure is closely related to the formation of stress fibers, where they locate at two distinct ends of stress fibers. The increase in the presence of stress fibers and focal adhesions has been stipulated to have differential effects on cell migration, with some saying that the increased number of FAs can lead to overt adherence and impairment in migration properties^78^. Here, we show that the knockout of *ACTBL2* influenced the formation of focal adhesions. We saw an increase in the nascent FAs number, but this was only demonstrated in normal conditions, and the same results were not obtained by LPA stimulation. This is surprising, as we saw an increase in the number of stress fibers after LPA stimulation, but this can mean more of a stabilization effect of the mature stress fibers, rather than the formation of nascent ones. Instead of that, we saw an increase in the number of mature FAs (α-Parvin staining), both in normal conditions and upon LPA stimulation.

Similarly to the findings presented here, we showed earlier that with the increase in the number of stress fibers, there was an increase in the formation of focal adhesions in cells devoid of β- or γ-actin^8^. Also, Bunnell et al. showed that in cells isolated from *ACTB* null mice, there was an increase in stress fibers and focal adhesions^72^. They also saw a correlation, where the increased number of FAs formation leads to impaired 2D migration, which can be seen in our study. The results obtained for CR-*ACTBL2* cells imply that actbl2-devoid cells exhibit mixed behavior compared to CR-*ACTB* and CR-*ACTG1* cells^8^. Opposite results could be partly seen in the case of overexpression of actbl2 in this study, with a decrease in FAs number, both nascent and mature, but only under physiological conditions. Upon LPA treatment, only the number of mature stress fibers was (slightly) significantly higher. There was, however, an increase in the mean area of the focal contacts in OE-*ACTBL2* cells upon PMA stimulation, with an overall decrease in their number, suggesting that the size and formation of FA could be differentially regulated and have varying effects on the cells ability to migrate. We did not see the opposite results in CR-*ACTBL2* clones upon PMA treatment.

All of the above indicate that the mechanisms regulating cell migration and adhesion need to be further studied. As there are so many interactions taking place, there is differential regulation of these processes when it comes to different actin isoforms. Altogether, our data suggest that actbl2 is a new player in the motility of melanoma cells, but manipulations with its level give slightly different outcomes than those observed for β- or γ-actin^8^. Again, this supports the notion that non-muscle actin isoforms differ in their properties, which is mirrored in their action at the cellular level^8,79^.

Overall, with this study, we were able to provide significant new knowledge in the field of actin isoforms, including identifying actbl2 as the seventh isoactin. We inferred their origin and evolution and showed relevant functional data concerning the poorly understood β-actin-like protein 2. Our results point to the importance of actbl2 in migration processes happening in the cell. Although this actin isoform is present at a very low level in melanoma cells, knocking it out significantly influences the cell functions. Moreover, we show that actbl2 has an impact on the formation of focal adhesion. This is of high importance to further study the signaling pathways controlling the most critical processes happening in the cell that concern actbl2. Additionally, studies on recombinant actbl2, β- and γ-actin should be designed to spot the differences in biochemical features between these three non-muscle actins. Our research had bridged the gap in knowledge of actbl2, as it has been shown before, that non-muscle isoactins were not equal in terms of cell motility.

## Methods

### Bioinformatic and phylogenetic analyses

#### Selection and alignment of actin sequences

To collect a comprehensive set of sequences similar to actbl2, we conducted local sensitive searches of the NCBI Reference Sequence Database RefSeq^80^ using BLASTP^81^, assuming word size = 2 and E-value < 0.001. This resulted in 13,694 sequences, which we subjected to clustering in CLANS (Cluster Analysis of Sequences)^82^. This software visualizes BLAST pairwise sequence similarities in either two-dimensional or three-dimensional space. Based on this approach, we separated a coherent set of 2033 sequences, the most closely related to actbl2. The sequences were aligned in MAFFT using the slow and accurate algorithm L-INS-i with 1,000 cycles of iterative refinement^83^. After the elimination of incomplete and fragmentary sequences, the set comprised 1540 sequences, of which 1441 were non-redundant. The alignment of these sequences with 376 sites was subjected to further studies. For each amino acid sequence, including its all identical versions, we downloaded from GenBank nucleotide sequences in the total number of 4179, which were aligned based on their amino acid alignment. After the elimination of redundant sequences, the final set consisted of 3554 sequences with 1128 alignment sites. Despite the removal of identical sequences from the sets, we retained information about the taxonomic affiliation of each sequence. The alignments were inspected in JalView^84^.

#### Phylogenetic analyses of actin sequences

Phylogenetic trees were inferred using a maximum likelihood approach in IQ-TREE^85^ and the Bayesian method in MrBayes^86^ based on the produced alignments. For the set of amino acid sequences, we applied the LG + F + R8 amino acid substitution model in IQ-TREE, as proposed by the ModelFinder program^87^, whereas for the nucleotide data set, we used three separate substitution models for three codon positions: SYM + R10 (the 1st position), GTR + F + R7 (the 2nd position), and SYM + ASC + R8 (the 3rd position), as indicated by the partition model of ModelFinder ^88^. In the tree search, we used the more thorough and slower NNI tree search, which considers all possible NNIs instead of only those similar to the previous NNI. To assess the significance of clades, we applied the Shimodara-Hasegawa-like approximate likelihood ratio test (SH-aLRT) with 10,000 replicates and non-parametric bootstrapping with 1000 replicates.

In MrBayes analyses, we used the mixed models of substitutions, rather than a fixed model to specify appropriate rate matrices across the ample parameter space^89^. However, we assumed a proportion of invariant sites and the gamma-distributed rate variation with five discrete rate categories to describe the heterogeneity rate across alignment sites, as proposed by the ProtTest^90^ for the amino acid alignment and by PartitionFinder^91^ for the nucleotide alignment. Thus, for the former set, we applied a mixed + I + Γ5 models and, for the latter, three separate models for three codon positions: mixed + I + Γ5 (the 1st position), mixed + I + Γ5 (the 2nd position), and mixed + Γ5 (the 3rd position). In the Bayesian approach, we executed two independent runs starting from random trees, each using 96 Markov chains. The trees were sampled every 100 generations for 25 million (for the amino acid set) or 43 million (for the nucleotide set) generations with 100 sample frequency. To compute a consensus tree, we selected trees from the last 10 million generations that reached the stationary phase and convergence. Phylogenetic trees were inspected and edited in FigTree^92^ and TreeGraph^93^.

Pairwise phylogenetic distances were calculated for each actin group. The distances were obtained from the MrBayes tree based on the amino acid alignment and were compared in the Kruskal–Wallis test with Dunn’s post-hoc test. In this posthoc test, the Benjamini–Hochberg method was applied for p-value correction to control the false discovery rate. P-values smaller than 0.05 were considered significant. The statistical analysis was performed in the R package (A language and environment for statistical computing, R Core Team, 2020, R Foundation for Statistical Computing, Vienna, Austria).

#### Profile analyses

The HMMER 3.3 package^94^ was applied to create profile hidden Markov models (HMMs) for individual seven groups of actins both at the amino acid and nucleotide levels. Initial profiles were constructed based on sequences that were closely related in phylogenetic trees, i.e., grouped in one cluster, with seven human actins: *ACTB*, *ACTG1*, *ACTA2*, *ACTA1*, *ACTC1*, *ACTG2*, and *ACTBL2* (Table S1). We conducted such analyses separately for sequences groups extracted from IQ-TREE and MrBayes phylogenetic trees. The profiles were used for scanning all sequences to classify them into the individual actin groups. A sequence was assigned to a given actin profile if it was the best hit for this sequence, and the score was more significant than the minimum value obtained for the given actin group. Finally, a sequence was assigned to one of the seven actin groups according to four criteria: its relationship to the human actins in two phylogenetic trees (IQ-TREE and MrBayes), as well as two assignments to profiles created on sequences extracted from these trees (Table S1). A given sequence was classified into an actin group if at least three criteria indicated this group, or this sequence was clustered with the actin group in two phylogenetic trees. The classification of sequences to the actin groups was superimposed into the presented phylogenetic trees. The selected amino acid sequences were used to create the final profile HMMs, which were visualized as logos in the Skylign tool, assuming information content above background^95^.

#### Phylogenetic analyses of profiles HMMs and consensus sequences

The pHMM-Tree software^96^ was applied to create a distance matrix between the amino acid actin profiles, which were aligned in PRC^97^. To depict the evolutionary relationships between the actin profiles, the phylogenetic trees were created based on the matrix in MEGA X^98^ using neighbor-joining and minimum evolution methods. For the amino acid profile of each actin group, we generated a majority-rule consensus sequence, consisting of amino acid residues present in the majority of sequences belonging to individual actin classes, which were aligned in MAFFT and used in phylogenetic analyses in IQ-TREE^85^, morePhyML^99^, MrBayes^86^, and PhyloBayes^100^. We used the LG + I model in IQ-TREE as proposed by ModelFinder as well as MtArt + F + Γ5 in (more)PhyML, mixed + I + Γ5 in MrBayes, and MtArt + F + Γ5 in PhyloBayes, according to ProtTest results. We applied the more thorough NNI tree search in IQ-TREE and the best heuristic search algorithm, (NNI + SPR) in (more)PhyML.

To assess the significance of tree branches, the SH-aLRT approach and non-parametric bootstrapping with 1000 replicates were used. In MrBayes, we applied two independent runs with four Markov chains. Trees were sampled every 100 generations for 10 million generations, and a posterior consensus was calculated for trees from the last 5,808,000 generations that reached the stationary phase and convergence. In PhyloBayes, two independent Markov chains were run for 100,000 generations, with one tree sampled for each generation. The last 50,000 trees from each chain were collected to compute the posterior consensus tree after reaching the convergence.

#### Testing tree topologies

Four tree topologies describing different relationships between seven groups of actins and obtained in the phylogenetic analyses of profiles, as well as alignments of 1441 amino acid sequences and seven consensus sequences, were compared using tree topology tests in IQ-TREE and Consel^101^ assuming 10,000,000 replicates. In the latter approach, site-wise log-likelihoods for the analyzed trees were calculated in PhyML. Moreover, the tree topologies were compared using Bayes Factor calculated in MrBayes based on the stepping-stone method estimating the mean marginal likelihood from two independent runs using four Markov chains, 50 steps of the sampling algorithm, and 10,000,000 generations of the MCMC simulation. All the analyses were conducted on the alignment of consensus sequences, assuming best-fitted substitution models for the given approach.

### Cell lines and culture conditions

Cell lines were cultured and stimulated with LPA or PMA, as described elsewhere^8^. A375 and Hs294T cell lines were obtained from the ATCC, WM1341D, and WM9 cell lines were from Rockland Immunochemicals, Inc., whereas SK-MEL-28 were bought at CLS Cell Lines Service GmbH. Cell lines were subcultured twice per week. Before Phorbol-12-Myristate-13-Acetate (PMA, Santa Cruz Biotechnology Inc.) or Lysophosphatidic Acid (LPA, Santa Cruz Biotechnology Inc.) treatment, the cells were starved for 24 h in medium without FBS (fetal bovine serum). Cells were stimulated with 1 µM LPA for 10 min or with 100 nM PMA for 5 min at 37 °C in 5% CO_2_.

### CRISPR/Cas9(D10A) inactivation of *ACTBL2* genes

A375 cells growing in a 35 mm plate were transfected accordingly to the manufacturer and with the help of Lipofectamine 2000 (Invitrogen) with a mixture of two plasmids (sc-415610-NIC, Santa Cruz Biotechnology Inc.) coding for Cas9 (D10A), two gRNA specific for a gene of interest [or scrambled gRNAs (sc-437281, Santa Cruz Biotechnology Inc.) in the case of control clones], puromycin resistance and GFP . gRNAs sequences for *ACTBL2* were 5′ccagggcgttatggtaggca3′ and 5′cgaggacgccctatcatgga3′. Information about the control gRNAs sequences is confidential, according to the manufacturer. The procedure of clones generation is described elsewhere^8^.

### Clonings and transfections

pLVX-hPGK-Puro and pLVX-hPGK-Puro-Actbl2 plasmids were obtained based on pLVX-IRES-Puro-Tβ4 plasmid described elsewhere^75^. Here, the CMV promoter present in the pLVX-IRES-Puro-Tβ4 plasmid was substituted with hPGK promoter using *Cla*I and *EcoR*I restriction sites. Primers 5′cgataagcttgggagttccgcgttttggggttgcgccttt3′ and 5′cggttcactaaacgagctcttggggagagaggtcggtg3′ were used when pLKO.1-puro plasmid was a template in PCR. Whereas when as a template served 101 bp DNA fragment – 5′agagctcgtttagtgaaccgtcagatcgcctggagacgccatccacgctgttttgacctccatagaagacaccgactctactagaggatctatttccggtg3′—we used following primers 5′atcaccgacctctctccccaagagctcgtttagtga3′ and 5′tttgtcagacatggtgaattcaccggaaatagatcc3′. Having two PCR products coding for hPGK and the spacer, we assembled the pLVX-hPGK-Puro-Tβ4 plasmid using the NEBuilder HiFi DNA Assembly Cloning Kit (New England BioLabs Inc.). Next, we cut out with the help of *EcoR*I and *BamH*I enzymes the sequence coding for Tβ4. The cut plasmid was ligated to obtain the “empty” control vector—pLVX-hPGK-Puro, or we cloned into it the actbl2 coding sequence with preserved 3′UTR region. PCR product was obtained based on A375 cells’ cDNA and the following primers: 5′tagaggatctatttccggtgaattcaccatgactgacaatgagctg3′ and 5′taggggggggggagggagaggggcgggatccatacaagtatgaagcaattttaatgtactgaaag3′. Next, it was cloned into the vector with the use of *EcoR*I and *BamH*I restriction sites to obtain pLVX-hPGK-Puro-Actbl2 plasmid. This cloning procedure was conducted with the NEBuilder HiFi DNA Assembly Cloning Kit (New England BioLabs Inc.).

For stable transfection, A375 cells growing in a 35 mm plate were transfected with the pLVX-hPGK-Puro-actbl2 plasmid coding for actbl2 under hPGK promoter and puromycin resistance with the help of Lipofectamine 2000, accordingly to the manufacturer. 24 h later, the medium was changed and replaced with the medium supplemented with 1 μg/ml of puromycin. Every 2–3 days, the medium was changed. Upon approximately two weeks, when the transfected cells formed colonies, they were isolated using glass cylinders and silicone grease. The clones were grown in the presence of 0.5 μg/ml puromycin containing medium.

For transient transfections, plasmids p3xHA-C1-actbl2 3′UTR, p3xHA-C1-β actin 3′UTR, and p3xHA-C1-γ actin 3′UTR were used. Using the primers: 5′gttatggatgatgatatcgccgcg3′and 5′gctaaggtgtgcacttttattcaac3′ (for β actin); 5′gttatggaagaagagatcgccgc 3′ and 5′gggttacggcagcacttttatttt3′ (for γ actin); 5′aaactcgagttatgactgacaatgagctgtc 3′ and 5′aaatctagaatacaagtatgaagcaat3′ (for actbl2); and cDNA of A375 cells as a template, we obtained PCR products, which were cloned into the p3xHA-C1 plasmid described elsewhere^102^. While cloning, we took advantage of *Xho*I and *Xba*I restriction sites. Sequences coding for actins were under CMV promoter.

The accuracy of all DNA constructs was verified by sequencing. Details concerning the generation of vectors are summarized in Table S4.

### RNAscope analysis

The *in situ* hybridization RNAscope 2.5 Chromogenic Assay (Advanced Cell Diagnostics) was performed strictly according to the manufacturer’s protocol. The cells were seeded in 8 well Nunc Lab-Tek II Chambered Coverglass chambers (ThermoFisher Scientific Scientific). 24 h later, upon reaching 70–80% confluency, the cell medium and the chambers were removed, and the cells were fixed in 10% Neutral Buffered Formalin (Sigma-Aldrich) for 30 min at RT. After washing three times in 1 X PBS, the cells were dehydrated using 50%, 70%, and 100% EtOH for 5 min, respectively. Lastly, the cells were left in 100% EtOH for 10 min and stored in 100% EtOH at − 20 °C until ready to use. On the day of the assay, the cells were rehydrated by incubation with 70% EtOH for 2 min, 50% EtOH for 2 min, and finally with 1 X PBS for 10 min. A hydrophobic barrier was created between distinct samples by using an Immedge hydrophobic barrier pen. From this point forward, all incubations were done in HybEZ Humidity Control Tray and HybEZ hybridization oven (steps with incubation at 40 °C). Next, the cells were treated with Hydrogen Peroxide for 10 min at RT. The slides were washed in 1 X PBS and treated with diluted Protease III (1:15 in 1 X PBS) for 10 min at RT. The slides were washed in PBS and ready for hybridization steps of the assay. The target probe (Actbl2), as well as the positive (PPIB) and negative (dapB) control probes, were added to designated samples and incubated for two h at 40 °C. After that time, the cells were washed for 2 min in 1 X Wash Buffer 2 times, and the first hybridization began by adding AMP 1 to cover the section entirely, and the slides were incubated for 30 min at 40 °C. The cells were washed and incubated with AMP 2 for 15 min at 40 °C, AMP 3 for 40 min at 40 °C, AMP 4 for 15 min at 40 °C, AMP 5 for 30 min at RT and AMP 6 for 15 min at RT. 2 washes separated each of the aforementioned steps in 1 X Wash Buffer for 2 min. For the detection of the signal, the Red B solution was diluted in the Red A solution at a 1:60 ratio and immediately added to the slides and incubated for 10 min at RT. After this step, the slides were washed with water, counterstained with 50% Hematoxylin for 2 min at RT, washed three times in water, and submerged into a 0.02% Ammonia water solution. After 10–15 s incubation, the slides were washed in water about three times, and the slides were dried in a hybridization oven for 20 min at 60 °C, after which they were dipped in xylene and mounted with the use of VectaMount Permanent mounting medium (Vector Laboratories). The slides were air-dried and analyzed using Leica TCS SP8 Confocal Laser Scanning Microscope, and Leica Application Suite X (LAS X) as FastRed dye also gives fluorescence.

### Invasion assay

The assay is described elsewhere^75^. Briefly, the test was performed using Transwell filters (BD Bioscience) coated with Matrigel (BD Bioscience). Cells that traversed the Matrigel layer and attached to the lower surface of the filter were fixed and stained with Hoechst 33342 and counted under a fluorescent microscope.

### Cell migration assays

To perform spontaneous migration assay, 1000 cells were seeded into wells of 96-well IncuCyte ImageLock plates. Time‐lapse photos were captured using IncuCyte Live Cell Analysis Imaging System for 72 h with a time interval of 2 h. For the evaluation of cellular trajectory, velocity and distance covered by single-cell Manual Tracking plug-in (ImageJ, F. Cordelieres, Institute Curie, Paris, France) were used. End-point directionality was calculated as the ratio between the straight-line displacement (distance to the origin, DTO) and the total path traveled by the cell (total distance, TD)^75^. For each parameter, 30 cells (3 clones) per group were analyzed.

To perform collective migration assay, cells were cultured into 96-well IncuCyte ImageLock plates. After 24 h, when the cells reached confluency, wounds in the cells monolayers were made in all wells simultaneously using Wound Maker. Then, the closure of the wound was followed using the IncuCyte Live Cell Analysis Imaging System. Images were taken every two h for 72 h and analyzed with IncuCyte software. The results were presented as a percent of scratch overgrown by cells over time.

### Quantitative polymerase chain reaction (qPCR) and RT-PCR

RNA was isolated with GenElute Mammalian Total RNA Miniprep Kit (Sigma Aldrich, RTN70). DNAse I (Sigma Aldrich, AMPD1-1KT) was used to remove gDNA contamination. Using High Capacity cDNA Reverse Transcription Kit (Thermo Fisher Scientific), following the manufacturer's instructions, reverse transcription was performed. Real-time PCR reactions were carried out using the PowerUp SYBR Green Master Mix (Thermo Fisher Scientific) following the seller’s recommendations on StepOnePlus Real-Time PCR Systems device (Applied Biosystems). The list of primers is provided in Table S5. The presented results were normalized against the expression of *HPRT1*. One-step qRT-PCR was performed accordingly to the kit’s manufacturer’s (Thermo Fisher Scientific) recommendation to check whether isolated RNA from melanoma cells is contaminated with genomic DNA. 0.25 μg of RNA was used as a template for every reaction; half of the samples lacked reverse transcriptase (RT-enzyme). Reactions were run in the StepOnePlus Real-Time PCR Systems device (Applied Biosystems).

### gDNA analysis

Genomic DNA was isolated with the help of a DNA Purification Kit (EurX ) according to the manufacturer’s instructions. PCR reaction was performed with Color Taq PCR Master Mix (2x) (EurX) and primers listed in Table S5 annealing to the sequences upstream and downstream from the sequences recognized by the guide RNAs coded by CRISPR/Cas9(D10A) plasmids. PCR products were analyzed in 2% (w/v) agarose gel in Tris–acetate–EDTA (TAE) buffer. PCR product length of clones with knockout of actin isoforms was compared to PCR product length of control clones.

To check if both alleles were changed in every CR-*ACTBL2* clone regarding sequence coding for actbl2 gDNA of clones was isolated and served as a template for a PCR reaction. Primers are listed in Table S4. The PCR products obtained with Phusion High-Fidelity DNA Polymerase (HF Buffer) (ThermoFisher Scientific Scientific) were cloned into pAcGFP-C1 plasmid linearized with *EcoR*I using NEBuilder HiFi DNA Assembly Master Mix (New England BioLabs Inc.). *E. coli* DH5 α strain was as next transformed with HiFi reactions. Plasmids from bacterial clones were isolated and screened with restriction reactions to find the correct clones. Finally, the selected plasmids were sent to sequencing analysis at Microsynth GmBH. The results are presented in Fig. S13.

### Immunocytochemistry and confocal microscopy

Cells were fixed on coverslips with 4% formaldehyde for 20 min, and then the permeabilization of cells was done with 0.1% Triton X-100 in PBS. After that, coverslips were incubated with 1% BSA in PBS for blocking of non-specific bindings. Incubation with appropriate primary antibodies was performed overnight at 4 °C, while incubation with secondary antibodies and Hoechst 33342 (Invitrogen) was performed at room temperature for 1 h. Secondary antibodies were used in conjugation with Alexa-Fluor dyes. For visualization of G-actin and F-actin, cells were stained with Alexa Fluor 594-labeled DNase I and Alexa Fluor 488- or 568-labeled or CruzFluor 350-labeled phalloidin, respectively. Dilutions for all antibodies used for our studies are listed in Table S6. After staining, coverslips were mounted on glass slides with Dako Mounting Medium (Agilent Technologies). Taking of photos and analysis of immunostained cells were performed with Leica TCS SP8 Confocal Laser Scanning Microscope and Leica Application Suite X (LAS X).

### STED microscopy

Cells cultured on glass coverslips and transfected or treated were fixed with 4% formaldehyde for 20 min and then permeabilized with 0.1% Triton X-100 in PBS or ice-cold acetone for six min., respectively. Upon blocking the coverslips for 30 min with 1% BSA in PBS, HA-actins were detected using rabbit anti-HA antibodies, and next secondary antibodies conjugated to the dye Alexa Fluor 647 (Invitrogen). F-actin was detected with incubation with phalloidin coupled to Star Red. Incubation steps with antibodies or phalloidin were carried out for one hour at RT. Abberior Mount Liquid was used to fix the coverslips on the microscopic slides. Dilutions of antibodies and dyes in blocking solution are listed in Table S6. Super-resolution STED imaging was performed with a STEDyCON (Abberior Instruments) mounted at the camera port of a NikonTi2-E upright microscope equipped with a 100 × objective (APO100x/1.4 Oil). Alexa Fluor 647 and Star Red were imaged with excitation at a wavelength of 640 nm and time-gated fluorescence detection between 650 and 720 nm. The STED laser had a wavelength of 775 nm and a pulse width of roughly 1–7 ns. The pinhole was set to 1.13 AU. The photos were further processed (black/white inversion) in Fiji software^103^.

### Western blot analysis

The cell lysates were prepared by scraping the cells on ice in cytoskeletal-bound protein extraction buffer (10 mM Tris–HCl pH 7.4, 100 mM NaCl, 1 mM EDTA, 1 mM EGTA, 1 mM NaF, 20 mM Na_4_P_2_O_7_, 2 mM Na_3_VO_4_, 1% Triton X-100, 10% glycerol, 0.1% SDS, 0.5% sodium deoxycholate) with addition of 1:100 protease inhibitor cocktail (Sigma-Aldrich). The protein concentration in lysates was established by using the Bradford protein assay. The preparation of lysates for electrophoresis was done using the 4 × Sample Loading Buffer (40% glycerol, 240 mM Tris–HCl pH 6.8, 8% SDS, 0,04% bromophenol blue, 5% β-mercaptoethanol). The samples containing 30 μg of protein were separated on 12.5% polyacrylamide gel by SDS-PAGE and transferred to nitrocellulose by semi-wet transfer. The rest of the procedure is described elsewhere^8^. The dilutions of antibodies are listed in Table S6. The densitometric analysis was performed using the Image Lab software (Bio-Rad). The bands were standardized to whole protein content in the analyzed lane (Ponceau S) and then normalized against the mean of protein expression in the control group.

### NGS analysis

RNA was isolated with the RNeasy Mini Kit (Qiagen) according to the manufacturer's recommendations. The total RNA concentration was measured using the NanoPhotometer P360 Spectrometer (Implen GmBH, Germany). The quality of RNA was determined by using an RNA 6000Nano LabChip Kit and Agilent Bioanalyser 2100 (Agilent, Palo Alto, CA, USA). The quality of reads was checked with FastQC software v0.11.7^104^. Raw reads were mapped to reference human genome GRCh38 from Ensembl^105^ using Hisat2 software v 2.1.^106^. To calculate the transcripts level, Cuffquant and Cuffmerge v. 2.2.1 software were used^107^ together with file GTF Homo-sapiens.GRCh38.94.gtf from the Ensembl base. Cuffmerge software was used with the following parameter—library-norm-method classic-fpkm to normalize expression values with the FPKM algorithm.

### F:G actin ratio assay

The procedure was performed as described elsewhere^8^. Actin stabilizing buffer was used to harvest and lyse the cells (50 mM PIPES pH 6,9; 50 mM NaCl; 5 mM MgCl_2_; 5 mM EGTA; 5% (v/v) glycerol; 0,1% Nonidet P40; 0,1% Triton X-100; 0,1% Tween 20; 0,1% 2- mercaptoethanol; 0,001% Antifoam C; the cocktail of protease inhibitors 1:100; 1 mM ATP). The cells were lysed with the help of a special pipette, i.e., Microman E (Gilson). Lysates were incubated for 10 min at 37 °C and centrifuged at 100–300 × g for 5 min. Supernatants were centrifuged for 60 min at 100,000 × g in Optima MAX-XP with a TLA-55 rotor to separate F-actin from G-actin. Supernatants with G-actin were collected, while pellets with F-actin were resuspended in the actin depolymerization solution (10 μM cytochalasin D in water, Santa Cruz Biotechnology Inc.) and incubated for one h with occasional vortexing. Isolated fractions were analyzed using Western Blotting. One-fifth of both fractions were loaded on the gel. To determine the F/G-actin ratio, densitometric analysis was done as it is described in the Western blot analysis section.

### Focal adhesion and cell area analysis

Analysis of focal adhesion formation was performed on the cells immunostained with α Parvin or VASP, which were cultured under different conditions (in the presence or absence of FBS, stimulated with LPA or PMA). The number of focal adhesions was manually counted with the usage of annotation tools of the LasX application. Data were presented as an FAs’ number per cell. Both areas of focal adhesions and the size of the cell area were measured automatically using ImageJ software. Data were presented as a mean cell’s or FAs’ area. Thirty cells per group were analyzed except for FAs area analysis, where all FAs detected in 9 from these cells were taken for analysis. It allowed us to the determination of correlation between FAs number and FAs area.

### Lamellipodium width and thickness analysis

The procedure was performed as described elsewhere^8^. For the analysis of lamellipodia, the cells were seeded onto glass coverslips, after which they were treated with 100 nM PMA for 5 min and stained with phalloidin-Alexa Fluor 488. The immunofluorescent staining was imaged on ZEISS LSM 510 confocal microscope (Zeiss), followed by analysis using software dedicated to this microscope. For each clone, 20 cells were analyzed. The width of the lamellipodium was measured by guiding a histogram through the lamellipodium using a ruler in the software. The thickness of the lamellipodium was calculated by guiding a histogram through the lamellipodium and reading the whole intensity of fluorescence through the lamellipodium.

### Stress fibers formation analysis

The cells were seeded onto glass coverslips 24 h before the assay. The next day, they were treated with 1 µM LPA for 10 min. After that, the cells were fixed, and immunofluorescent staining was performed according to the protocol described in the previous section. The imaging was done using the ZEISS LSM 510 confocal microscope (Zeiss) and analysis performed in software dedicated to this microscope. The number of stress fibers per cell was assessed by guiding a histogram through the width of the cell containing thick stress fibers and identifying the number of fluorescent values that were higher or equal to 75% of the highest fluorescence peak. The analysis was done on 20 cells per clone.

### Gelatin digestion assay

The procedure was performed as described elsewhere^8^. The purpose of the experiment was to assess the number of active invadopodia digesting fluorescently-labeled gelatin in cells. Poly-L-Lysine coated coverslips (Corning) placed in a 24 well plate and rinsed with 1 × PBS were fixed by incubation with 0.5% glutaraldehyde for 15 min at RT, followed by another washing with PBS. The coverslips were immediately placed on a drop of gelatin-fluorescein (Invitrogen) and incubated in the dark for 10 min at RT. To quench residual glutaraldehyde, coverslips were incubated in 5 mg/mL sodium borohydride solution at RT, followed by washing with PBS. Before seeding the cells, the coverslips were rinsed with a cold cell culture medium. Thirty thousand cells were seeded per well, onto the side of coverslip coated with gelatin-fluorescein in a 24 well plate in a full cell culture medium and kept at 37 °C, 5% CO_2_ for 12 h before fixing with 4% FA. The cells were stained using phalloidin-Alexa Fluor 568 and Hoechst 33342 (Invitrogen) to visualize F-actin and cell nuclei, respectively. The images were taken on a confocal microscope. The number of invadopodia and the area of gelatin digestion was estimated by using the ImageJ software. The total number of cells per clone, which were analyzed, was 10.

### Statistical analysis

Statistical analysis and graphing were performed using GraphPad Prism 8 (GraphPad Software Inc.) as described elsewhere^8^. Three clones per group were analyzed for each experiment as the biological replicates. First, the normality of data distribution was determined with Shapiro–Wilk's or D'Agostino-Pearson normality tests. The significance levels were *p* < 0.05 (*), *p* < 0.01 (**), *p* < 0.001 (***) and *p* < 0.0001 (****). Parametric or nonparametric types of unpaired Student’s t-test or ANOVA (one-way or two-way) tests were used for the analysis of statistical data significances. Data were presented as means ± standard deviations (SD) or ± standard of the mean (SEM).

## Supplementary Information

Supplementary Information.

## Abbreviations

ABPs: Actin binding proteins
*ACTA1*: Gene coding for α skeletal actin
*ACTA2*: Gene coding for α smooth actin
*ACTB*: Gene coding for β non-muscle actin
*ACTBL2*: Gene coding for actbl2 (β-actin-like protein 2)
actbl2: β-actin-like protein 2; the product of *ACTBL2*
*ACTC1*: Gene coding for α cardiac actin
*ACTG1*: Gene coding for γ non-muscle actin
*ACTG2*: Gene coding for γ smooth actin
CR-*ACTBL2*: Cells with knocked out *ACTBL2*
CR-CTRL: Control cells
FA: Focal adhesion
F-actin: Fetal bovine serum
FBS: Filamentous actin
G-actin: Globular actin
gDNA: Genomic DNA
LPA: Lysophosphatidic Acid
nFA: Nascent focal adhesion
OE-*ACTBL2*: Cells with actbl2 overexpression
OE-CTRL: Control cells
PMA: Phorbol-12-Myristate-13-Acetate
SH-aLRT: Shimodara-Hasegawa-like approximate likelihood ratio test
