# Supplementary material for: The origin of the expressed retrotransposed gene ACTBL2 and its influence on human melanoma cells’ motility and focal adhesion formation
Source: Sci Rep. 2021 Feb 8;11:3329. doi: 10.1038/s41598-021-82074-x (PMC7870945; doi:10.1038/s41598-021-82074-x)
Supplement: Supplementary file 1 — Supplementary Information. [file 41598_2021_82074_MOESM1_ESM.pdf]

THE ORIGIN OF THE EXPRESSED RETROTRANSPOSED GENE *ACTBL2* AND ITS INFLUENCE ON HUMAN MELANOMA CELLS' MOTILITY AND FOCAL ADHESION FORMATION

Natalia Malek[1^], Aleksandra Michrowska[1^], Ewa Mazurkiewicz[1], Ewa Mrówczyńska[1], Paweł Mackiewicz[2], Antonina J. Mazur[1*]

[1]Department of Cell Pathology, Faculty of Biotechnology, University of Wroclaw, Poland

[2]Department of Bioinformatics and Genomics, Faculty of Biotechnology, University of Wroclaw, Poland

^Authors contributed equally to the manuscript

***Corresponding author:**

Dr. Antonina Joanna Mazur, DSc
antonina.mazur@uwr.edu.pl
Department of Cell Pathology
Faculty of Biotechnology
University of Wroclaw
ul. Joliot-Curie 14a
50-383 Wrocław
tel. + 48 71 37 56 206

Fig. S1. Cluster analysis of 13,694 actin homologs conducted in CLANS software. Individual sequences are represented by vertices. Edges connected these vertices (shown in the top panel) reflect attractive forces proportional to the negative logarithm of E-value calculated for the high scoring segment pairs (HSPs). The gray shade intensity of the connections is proportional to these forces. Subsequent panels show an enlarged part of a previous plot. The cluster of sequences closely related to actbl2 and used in this study is indicated by the red circle.

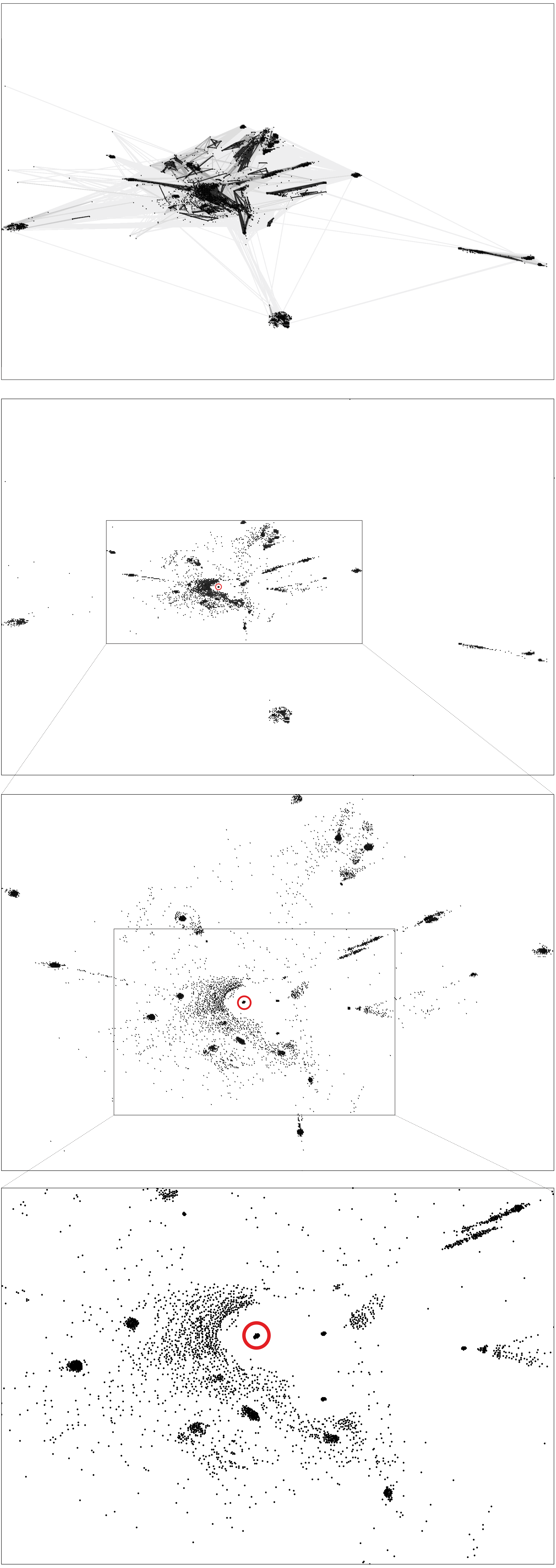

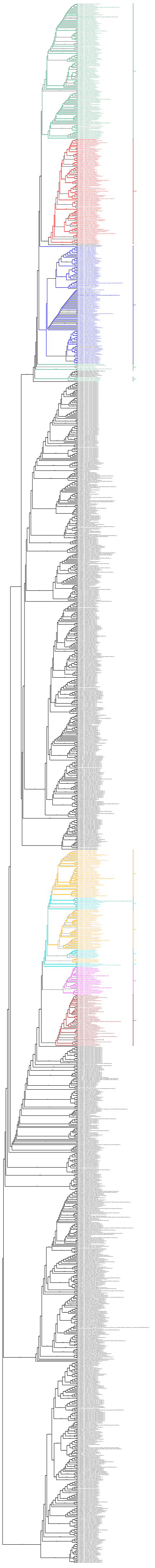

Fig. S2. Cladogram obtained in IQ-TREE showing relationships between 1441 amino acid sequences of actins and their relatives. Seven groups of actins homologous to human sequences: ACTB, ACTG1, ACTA2, ACTA1, ACTC1, ACTG2 and ACTBL2 were indicated in different colours. The human sequences or their identical homologs were bolded. The affiliation to main taxonomic groups was shown. The number of identical sequences for those presented in the tree is included in parentheses. Numbers at nodes correspond to support values calculated by aLRT based on a Shimodaira-Hasegawa-like procedure (before the slash) and bootstrap analysis (after the slash). Values lower than 50 were omitted or indicated by a dash "–".

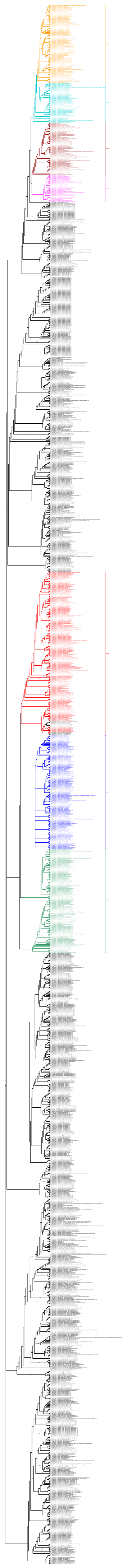

Fig. S3. Cladogram obtained in MrBayes showing relationships between 1441 amino acid sequences of actins and their relatives. Seven groups of actins homologous to human sequences: ACTB, ACTG1, ACTA2, ACTA1, ACTC1, ACTG2 and ACTBL2 were indicated in different colours. The human sequences or their identical homologs were bolded. The affiliation to main taxonomic groups was shown. The number of identical sequences for those presented in the tree is included in parentheses. Numbers at nodes correspond to posterior probabilities. Values lower than 0.5 were omitted.

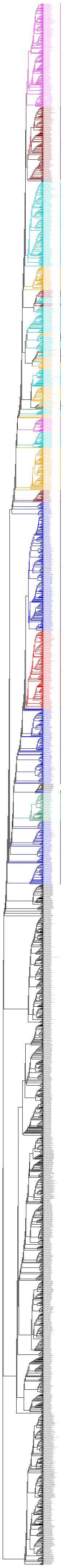

Fig. S4. Cladogram obtained in IQ-TREE showing relationships between 3554 nucleotide sequences of actins and their relatives. Seven groups of actins homologous to human sequences: ACTB, ACTG1, ACTA2, ACTA1, ACTC1, ACTG2 and ACTBL2 were indicated in different colours. The human sequences or their identical homologs were bolded. The affiliation to main taxonomic groups was shown. The number of identical sequences for those presented in the tree is included in parentheses. Numbers at nodes correspond to support values calculated by aLRT based on a Shimodaira-Hasegawa-like procedure (before the slash) and bootstrap analysis (after the slash). Values lower than 50 were omitted or indicated by a dash "–".

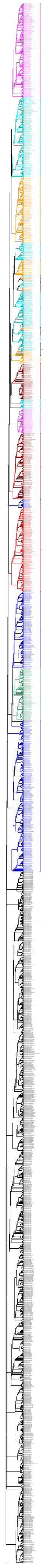

Fig. S5. Cladogram obtained in MrBayes showing relationships between 3554 nucleotide sequences of actins and their relatives. Seven groups of actins homologous to human sequences: ACTB, ACTG1, ACTA2, ACTA1, ACTC1, ACTG2 and ACTBL2 were indicated in different colours. The human sequences or their identical homologs were bolded. The affiliation to main taxonomic groups was shown. The number of identical sequences for those presented in the tree is included in parentheses. Numbers at nodes correspond to posterior probabilities. Values lower than 0.5 were omitted.

Fig. S6. Logos of profile HMMs for seven actin groups. The letter height in the given position corresponds to information content above background, i.e. only positive-scoring amino acids were included in the stack. The height of the stack was subdivided according to the relative probabilities of these positive scoring amino acids. Columns including non-conserved amino acid substitutions were indicted by red boxes.

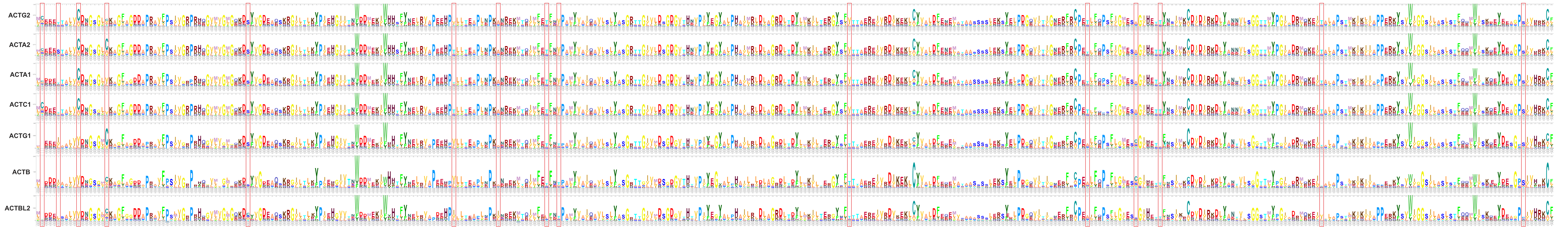

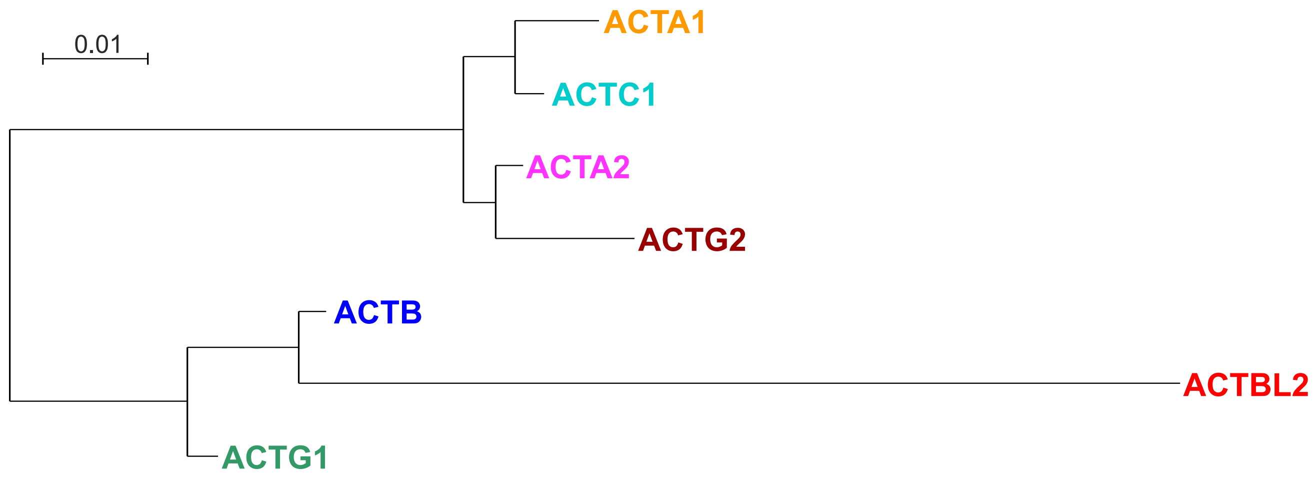

Fig. S7. Phylogram obtained in morePhyML showing relationships between consensus sequences of seven acting groups.

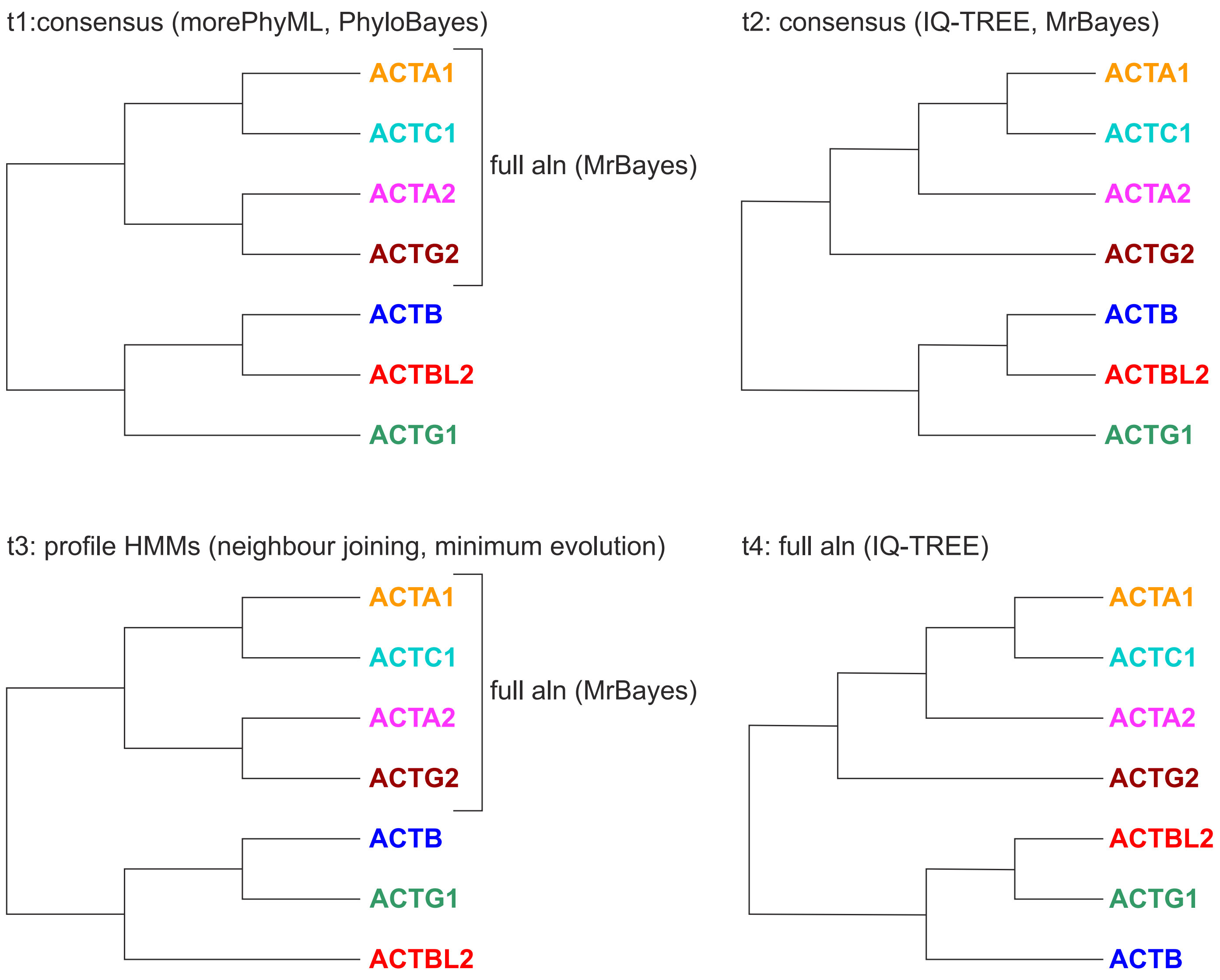

Fig. S8. Tree topologies obtained from various approaches and data sets: consensus sequences analyzed in morePhyML, PhyloBayes, IQ-TREE and MrBayes, distance matrices between amino acid profile HMMs analyzed by neighbor-joining and minimum evolution methods, as well as the full alignment of 1441 amino acid sequences (full aln) analyzed in MrBayes and IQ-TREE.

```
alpha skeletal  MCDEDETTALVCDNGSGLVKAGFAGDDAPRAVFPSIVGRPRHQGVMVGMGQKDSYVGDEAQSKRG
alpha cardiac   MCDDEETTALVCDNGSGLVKAGFAGDDAPRAVFPSIVGRPRHQGVMVGMGQKDSYVGDEAQSKRG
alpha smooth    MCEEEDSTALVCDNGSGLCKAGFAGDDAPRAVFPSIVGRPRHQGVMVGMGQKDSYVGDEAQSKRG
gamma smooth    MC-EEETTALVCDNGSGLCKAGFAGDDAPRAVFPSIVGRPRHQGVMVGMGQKDSYVGDEAQSKRG
beta            M--DDDIAALVVDNGSGMCKAGFAGDDAPRAVFPSIVGRPRHQGVMVGMGQKDSYVGDEAQSKRG
gamma           M--EEEIAALVIDNGSGMCKAGFAGDDAPRAVFPSIVGRPRHQGVMVGMGQKDSYVGDEAQSKRG
actbl2          MT-DNELSALVVDNGSGMCKAGFGGDDAPRAVFPSMIGRPRHQGVMVGMGQKDCYVGDEAQSKRG
                *   ::: :*** *****: ****.***********::****************.***********

alpha skeletal  ILTLKYPIEHGIITNWDDMEKIWHHTFYNELRVAPEEHPTLLTEAPLNPKANREKMTQIMFETFN
alpha cardiac   ILTLKYPIEHGIITNWDDMEKIWHHTFYNELRVAPEEHPTLLTEAPLNPKANREKMTQIMFETFN
alpha smooth    ILTLKYPIEHGIITNWDDMEKIWHHSFYNELRVAPEEHPTLLTEAPLNPKANREKMTQIMFETFN
gamma smooth    ILTLKYPIEHGIITNWDDMEKIWHHSFYNELRVAPEEHPTLLTEAPLNPKANREKMTQIMFETFN
beta            ILTLKYPIEHGIVTNWDDMEKIWHHTFYNELRVAPEEHPVLLTEAPLNPKANREKMTQIMFETFN
gamma           ILTLKYPIEHGIVTNWDDMEKIWHHTFYNELRVAPEEHPVLLTEAPLNPKANREKMTQIMFETFN
actbl2          VLTLKYPIEHGVVTNWDDMEKIWYHTFYNELRVAPDEHPILLTEAPLNPKINREKMTQIMFEAFN
                :**********::**********:*:*********:*** ********** ***********:**

alpha skeletal  VPAMYVAIQAVLSLYASGRTTGIVLDSGDGVTHNVPIYEGYALPHAIMRLDLAGRDLTDYLMKIL
alpha cardiac   VPAMYVAIQAVLSLYASGRTTGIVLDSGDGVTHNVPIYEGYALPHAIMRLDLAGRDLTDYLMKIL
alpha smooth    VPAMYVAIQAVLSLYASGRTTGIVLDSGDGVTHNVPIYEGYALPHAIMRLDLAGRDLTDYLMKIL
gamma smooth    VPAMYVAIQAVLSLYASGRTTGIVLDSGDGVTHNVPIYEGYALPHAIMRLDLAGRDLTDYLMKIL
beta            TPAMYVAIQAVLSLYASGRTTGIVMDSGDGVTHTVPIYEGYALPHAILRLDLAGRDLTDYLMKIL
gamma           TPAMYVAIQAVLSLYASGRTTGIVMDSGDGVTHTVPIYEGYALPHAILRLDLAGRDLTDYLMKIL
actbl2          TPAMYVAIQAVLSLYASGRTTGIVMDSGDGVTHIVPIYEGYALPHAILRLDLAGRDLTDYLMKIL
                .***********************:******** ************:*****************

alpha skeletal  TERGYSFVTTAEREIVRDIKEKLCYVALDFENEMATAASSSSLEKSYELPDGQVITIGNERFRCP
alpha cardiac   TERGYSFVTTAEREIVRDIKEKLCYVALDFENEMATAASSSSLEKSYELPDGQVITIGNERFRCP
alpha smooth    TERGYSFVTTAEREIVRDIKEKLCYVALDFENEMATAASSSSLEKSYELPDGQVITIGNERFRCP
gamma smooth    TERGYSFVTTAEREIVRDIKEKLCYVALDFENEMATAASSSSLEKSYELPDGQVITIGNERFRCP
beta            TERGYSFTTTAEREIVRDIKEKLCYVALDFEQEMATAASSSSLEKSYELPDGQVITIGNERFRCP
gamma           TERGYSFTTTAEREIVRDIKEKLCYVALDFEQEMATAASSSSLEKSYELPDGQVITIGNERFRCP
actbl2          TERGYNFTTTAEREIVRDVKEKLCYVALDFEQEMVRAAASSSPERSYELPDGQVITIGNERFRCP
                *****.*.**********:***********:**. **:*** *:********************

alpha skeletal  ETLFQPSFIGMESAGIHETTYNSIMKCDIDIRKDLYANNVMSGGTTMYPGIADRMQKEITALAPS
alpha cardiac   ETLFQPSFIGMESAGIHETTYNSIMKCDIDIRKDLYANNVLSGGTTMYPGIADRMQKEITALAPS
alpha smooth    ETLFQPSFIGMESAGIHETTYNSIMKCDIDIRKDLYANNVLSGGTTMYPGIADRMQKEITALAPS
gamma smooth    ETLFQPSFIGMESAGIHETTYNSIMKCDIDIRKDLYANNVLSGGTTMYPGIADRMQKEITALAPS
beta            EALFQPSFLGMESCGIHETTFNSIMKCDVDIRKDLYANTVLSGGTTMYPGIADRMQKEITALAPS
gamma           EALFQPSFLGMESCGIHETTFNSIMKCDVDIRKDLYANTVLSGGTTMYPGIADRMQKEITALAPS
actbl2          EAIFQPSFLGIESSGIHETTFNSIMKCDVDIRKDLYANTVLSGGSTMYPGIADRMQKEIITLAPS
                *::*****:*:**.******.*******:*********.*:***:************** :****

alpha skeletal  TMKIKIIAPPERKYSVWIGGSILASLSTFQQMWITKQEYDEAGPSIVHRKCF
alpha cardiac   TMKIKIIAPPERKYSVWIGGSILASLSTFQQMWISKQEYDEAGPSIVHRKCF
alpha smooth    TMKIKIIAPPERKYSVWIGGSILASLSTFQQMWISKQEYDEAGPSIVHRKCF
gamma smooth    TMKIKIIAPPERKYSVWIGGSILASLSTFQQMWISKPEYDEAGPSIVHRKCF
beta            TMKIKIIAPPERKYSVWIGGSILASLSTFQQMWISKQEYDESGPSIVHRKCF
gamma           TMKIKIIAPPERKYSVWIGGSILASLSTFQQMWISKQEYDESGPSIVHRKCF
actbl2          TMKIKIIAPPERKYSVWIGGSILASLSTFQQMWISKQEYDEAGPPIVHRKCF
                **********************************:* ****:**.*******
```

HQGVMVGMGQKDCYVGDEAQSK - Peptide detected in following studies: [19], [26] (MVGMGQKDCYVGD), [31], [33], [34], [37], [43] (DCYVGDEAQSK)

YPIEHGVVTNWDDMEK - Peptide detected in following studies: [24], [31], [32], [33], [36], [37]

IWYHTFYNELR - Peptide detected in following studies: [20], [22], [23], [28], [30], [31], [33], [34], [35], [36], [37], [38], [39]

VAPDEHPILLTEAPLNPK - Peptide detected in following studies: [9], [22], [23], [25], [27], [28], [29], [33], [39]

DLYANTVLSGGSTMYPGIADR - Peptide detected in following studies: [33], [41], [42]

Fig. S9. Sequences alignment of human typical actins and actbl2. Amino acid sequence alignment [MAFFT L-INS-i (v7.122b) [1]] of 6 classical actin isoforms and the new actbl2 isoform. The semicolon indicates conserved substitutions and dot semi-conserved substitutions in a given column. In microfilaments, one actin monomer forms contacts with four adjacent monomers, and in these interactions, the following residues take part: 322-325 interact with 243-245; 286-289 with 202-204; 166-169 and 375 with 41-45 (blue rectangles). The interactions along the genetic helix are between residues 110-112 and 195-197 (grey rectangles). All mentioned residues except 110-112 and 375 are located on the "back" surface of actin. The loop (a hydrophobic "plug") in 264-273 residues contacts two other actin monomers of the opposing strand [2] (green rectangle). Actin signatures I and II are highlighted in red (I - residues 54-64) and blue (II – residues 357-365), whereas the actin-like protein signature is highlighted in green (residues 105-117). Unique peptides of actbl2 identified by MS analysis in several proteomic studies are marked. References' numbers refer to the references' list in the main manuscript.

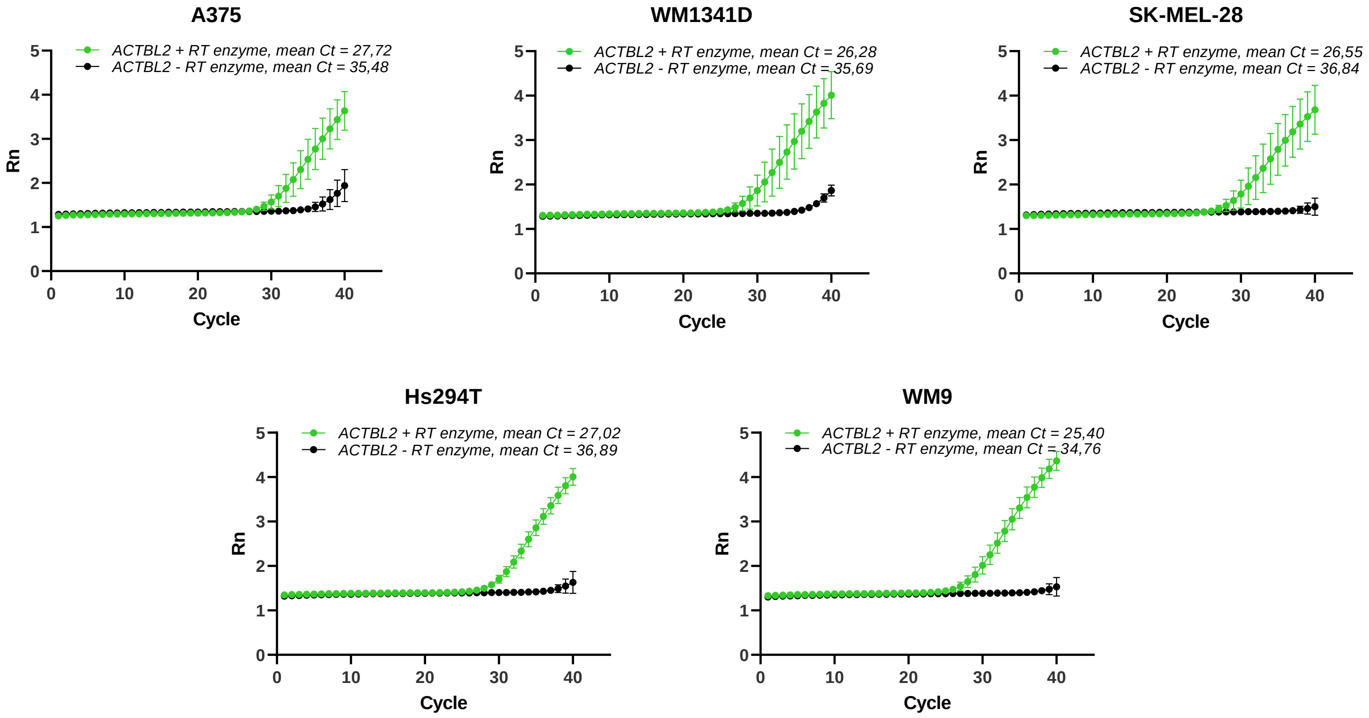

Fig. S10. Quantitative OneStep qPCR amplification curves for *ACTBL2*. As templates served 250 ng of RNA of 5 melanoma cell lines. (n=3). Results are expressed as the mean ±SD.

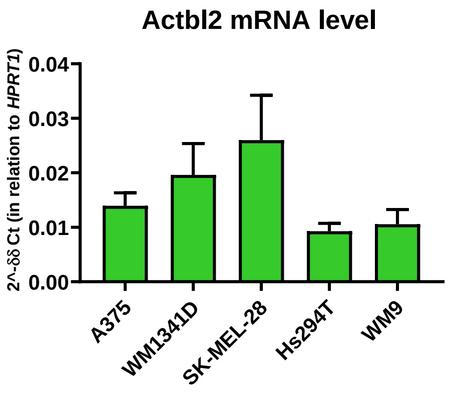

Fig. S11. Quantitative RT-PCR analysis of *ACTBL2* expression level in melanoma cell lines. cDNAs of 5 melanoma cell lines served as templates for the reaction. Results were normalized against the *HPRT1* gene (n=3). Results are expressed as the mean ±SD.

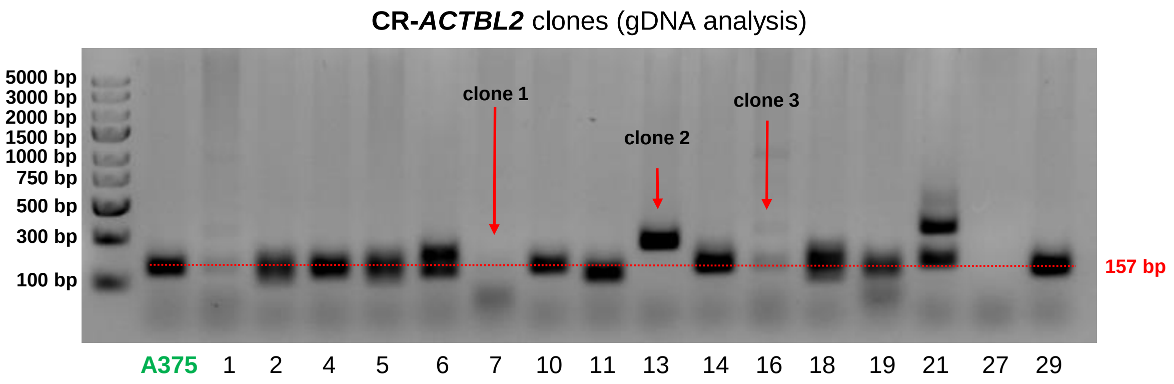

Fig. S12. Analysis of gDNA of obtained clones devoid of actbl2. Upon isolation of gDNA from clones, PCR reactions were performed with appropriate starters and 100 ng of gDNA as a template. Products of reactions were analyzed in 2% TAE agarose gel. bp – base pairs.

```
CR-CTRL c.1       cttgtcctgttcactcgtctggaagatctcagccagcaccatgactgacaatgagctgtc
CR-ACTBL2 c.1_1 cttgtcctgttcactcgtctggaagatctcagccagcaccatgactgacaatgagctgtc
CR-ACTBL2 c.1_2 cttgtcctgttcactcgtctggaagatctcagccagcaccatgactgacaatgagctgtc
CR-ACTBL2 c.2_1 cttgtcctgttcactcgtctggaagatctcagccagcaccatgactgacaatgagctgtc
CR-ACTBL2 c.2_2 cttgtcctgttcactcgtctggaagatctcagccagcaccatgactgacaatgagctgtc
CR-ACTBL2 c.3_1 cttgtcctgttcactcgtctggaagatctcagccagcaccatgactgacaatgagctgtc
CR-ACTBL2 c.3_2 cttgtcctgttcactcgtctggaagatctcagccagcaccatgactgacaatgagctgtc
                ************************************************************

CR-CTRL c.1       tgccttggtagtggataatgggtcagggatgtgcaaggcaggctttggtggtgacgatgc
CR-ACTBL2 c.1_1 tgccttggtagtggataatgggtcagggatgtgcaaggcaggctttggtggtgacgatgc
CR-ACTBL2 c.1_2 tgccttggtagtggataatgggtcagggatgtgcaaggcaggctttggtggtgacgatgc
CR-ACTBL2 c.2_1 tgccttggtagtggataatgggtcagggatgtgcaaggcaggctttggtggtgacgatgc
CR-ACTBL2 c.2_2 tgccttggtagtggataatgggtcagggatgtgcaaggcaggctttggtggtgacgatgc
CR-ACTBL2 c.3_1 tgccttggtagtggataatgggtcagggatgtgcaaggcaggctttggtggtgacgatgc
CR-ACTBL2 c.3_2 tgccttggtagtggataatgggtcagggatgtgcaaggcaggctttggtggtgacgatgc
                ************************************************************

CR-CTRL c.1       cccccgggctgtgttcccctccatgatagggcgtcctcgacaccagggcgttatgg----
CR-ACTBL2 c.1_1 cccccgggctgtgttcccctccatgatagggcgtcctc----------------------
CR-ACTBL2 c.1_2 cccccgggctgtgttcccctccatgatagggcgtcctc----------------------
CR-ACTBL2 c.2_1 cccccgggctgtgttcccatgggccatggg------------cccatggccatgg-----
CR-ACTBL2 c.2_1 cccccgggctgtgttcccctccatgatagggcgtcctcgacaccagggcgttatgatgat
CR-ACTBL2 c.3_1 cccccgggctgtgttcccctccatgatagggcgtcctcgacaccagggcgttatgatgat
CR-ACTBL2 c.3_2 cccccgggctgtgttcccctccatgatagggcgtcctcgacaccagggcgttatggtgag
                ****************** *      ** **

CR-CTRL c.1       ------------------------------------------------------------
CR-ACTBL2 c.1_1 ------------------------------------------------------------
CR-ACTBL2 c.1_2 ------------------------------------------------------------
CR-ACTBL2 c.2_1 ------------------------------------------------------------
CR-ACTBL2 c.2_2 aggacaccagggcgttatga----------tgataggacaccagggcgttatgatgatag
CR-ACTBL2 c.3_1 aggacaccagggcgttatga----------tgataggacaccagggcgttatgatgatag
CR-ACTBL2 c.3_2 gagctggtttttgaaaggatcaacaaaattgatagaccgctagcaagactaataaagaa

CR-CTRL c.1       ---------------------------------------------------------tag
CR-ACTBL2 c.1_1 ------------------------------------------------------------
CR-ACTBL2 c.1_2 ------------------------------------------------------------
CR-ACTBL2 c.2_1 ----------------------------------------------------------gc
CR-ACTBL2 c.2_2 gacaccagggcgttatgatgataggacaccagggcgttatgaccagggcgttattggtag
CR-ACTBL2 c.3_1 gacaccagggcgttatgatgataggacaccagggcgttatgaccagggcgttattggtag
CR-ACTBL2 c.3_2 aaaaagagagaagaatcaaatagacacaataaaaaatgataaaggggatatcaccaccga

CR-CTRL c.1       gcatg-ggccagaaggactgctacgtgggagatgaggctcagagcaagagaggcgtcctg
CR-ACTBL2 c.1_1 ----------------gactgctacgtgggagatgaggctcagagcaagagaggcgtcctg
CR-ACTBL2 c.1_2 ----------------gactgctacgtgggagatgaggctcagagcaagagaggcgtcctg
CR-ACTBL2 c.2_1 ccatg-ggccagaaggactgctacgtgggagatgaggctcagagcaagagaggcgtcctg
CR-ACTBL2 c.2_2 gcatg-ggccagaaggactgctacgtgggagatgaggctcagagcaagagaggcgtcctg
CR-ACTBL2 c.3_1 gcatg-ggccagaaggactgctacgtgggagatgaggctcagagcaagagaggcgtcctg
CR-ACTBL2 c.3_2 tcccacaatgataaggactgctacgtgggagatgaggctcagagcaagagaggcgtcctg
                                ********************************************
```

Fig. S13. Analysis of gDNA of obtained clones devoid of actbl2 production to check if ORF was shifted for every allele. Upon isolation of gDNA from clones, PCR reactions were performed with appropriate starters and gDNA as a template. Products were then cloned into the pAcGFP-C1 plasmid and selected plasmid clones were sequenced. The allele with the long insertion but no shift in ORF for actbl2 is highlighted in red. The other alleles have shifted ORFs in the region coding for actbl2. The codon start was shown in green. The star indicates identical nucleotide in given position for every allele.

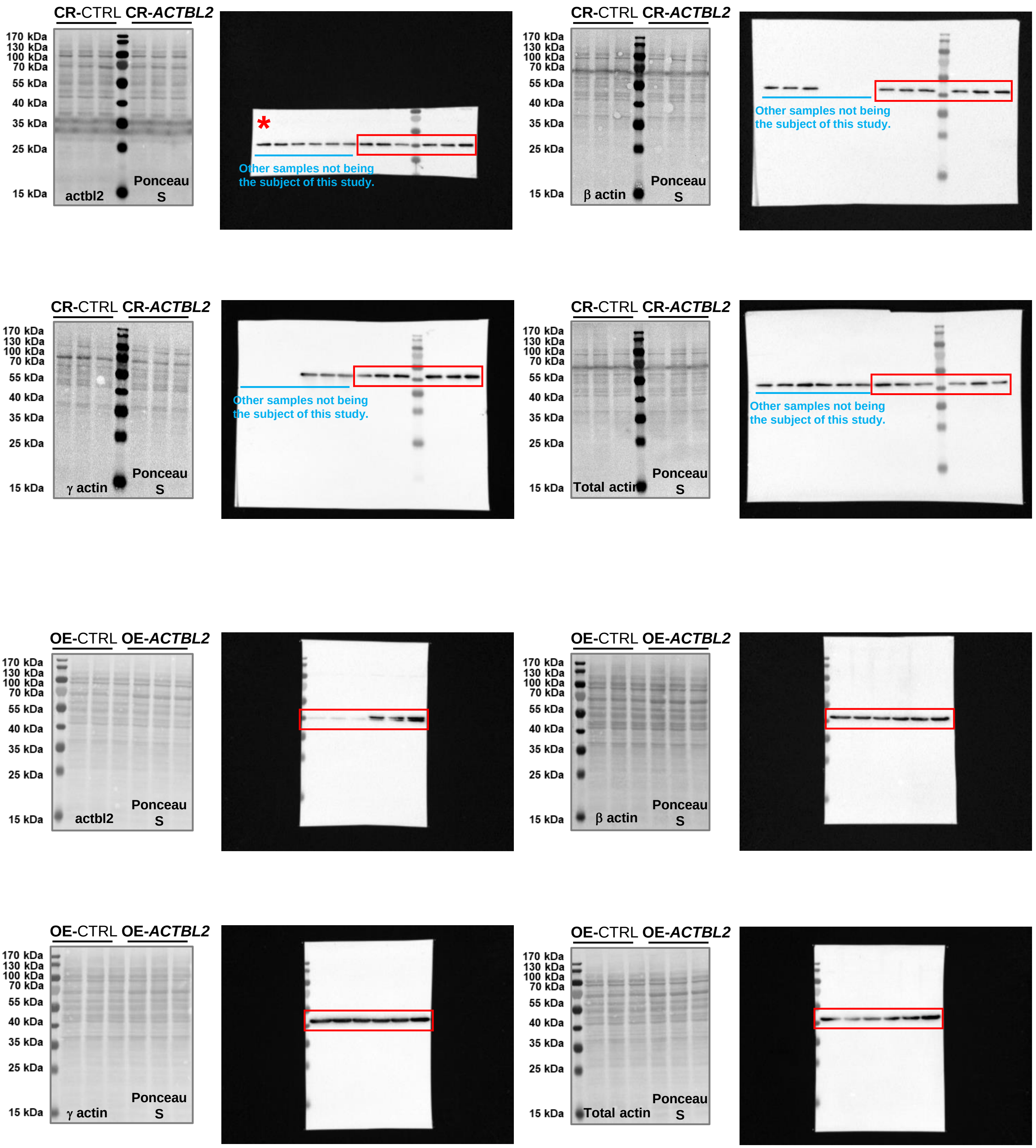

Fig. S14. Corresponding Ponceau S stainings of the membranes shown in Figure 6. Additionally uncroped membranes are shown. * The membrane was cut, because the upper and lower parts of the membrane were used for the reblotting to detect proteins of low abundance. The areas of the membranes shown in the Fig. 6 are marked here by red rectangles.

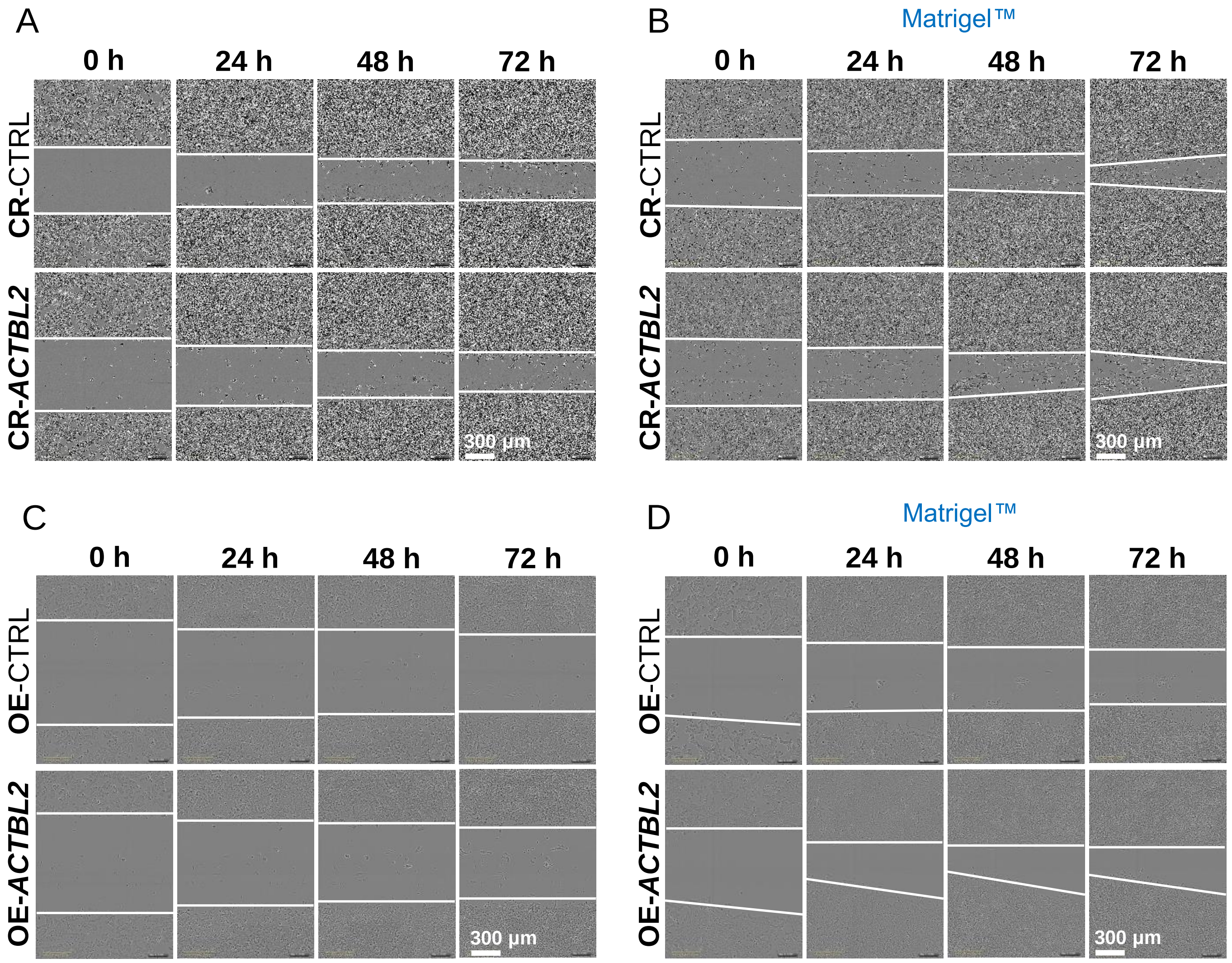

Fig. S15. Representative pictures of cells migrating collectively recorded over 72 h for studied cells (n=3). Results from collective migration are shown in Fig. 7.

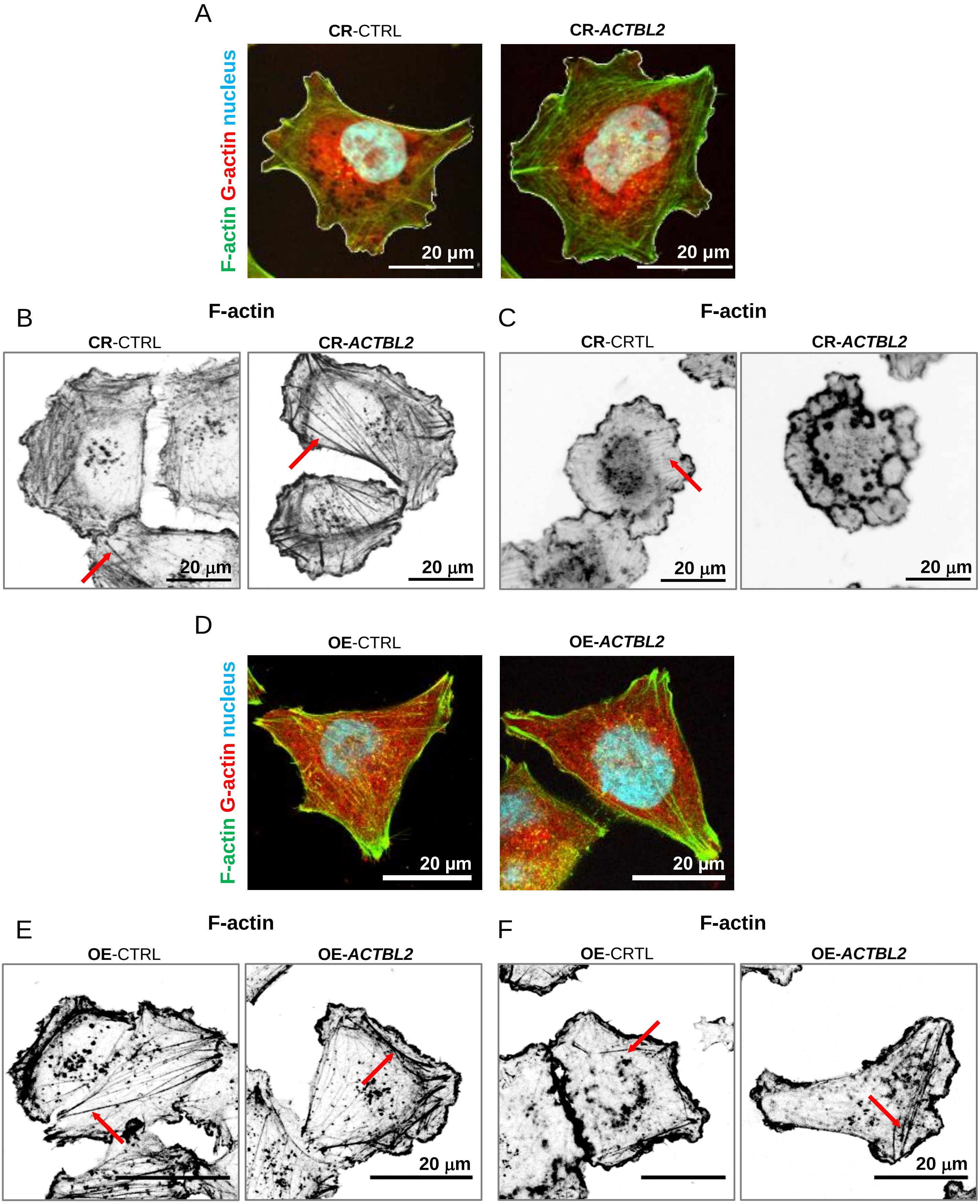

Fig. S16. The cells were grown under control conditions (the cells were grown in the presence of FBS) **(A, D)** or were treated with 1 mM LPA for 10 min. **(B, E)** or 100 mM PMA for 5 min. **(C, F)**. The cells were fixed and stained with either solely phalloidin-Alexa Fluor 488 (B, C, E, and F) or with phalloidin-Alexa Fluor 488, DNase I–Alexa Fluor 594 and Hoechst 33342 (A and D) to detect solely F-actin or F-actin, G-actin and cell nucleus, respectively. Red arrows point at stress fibers.

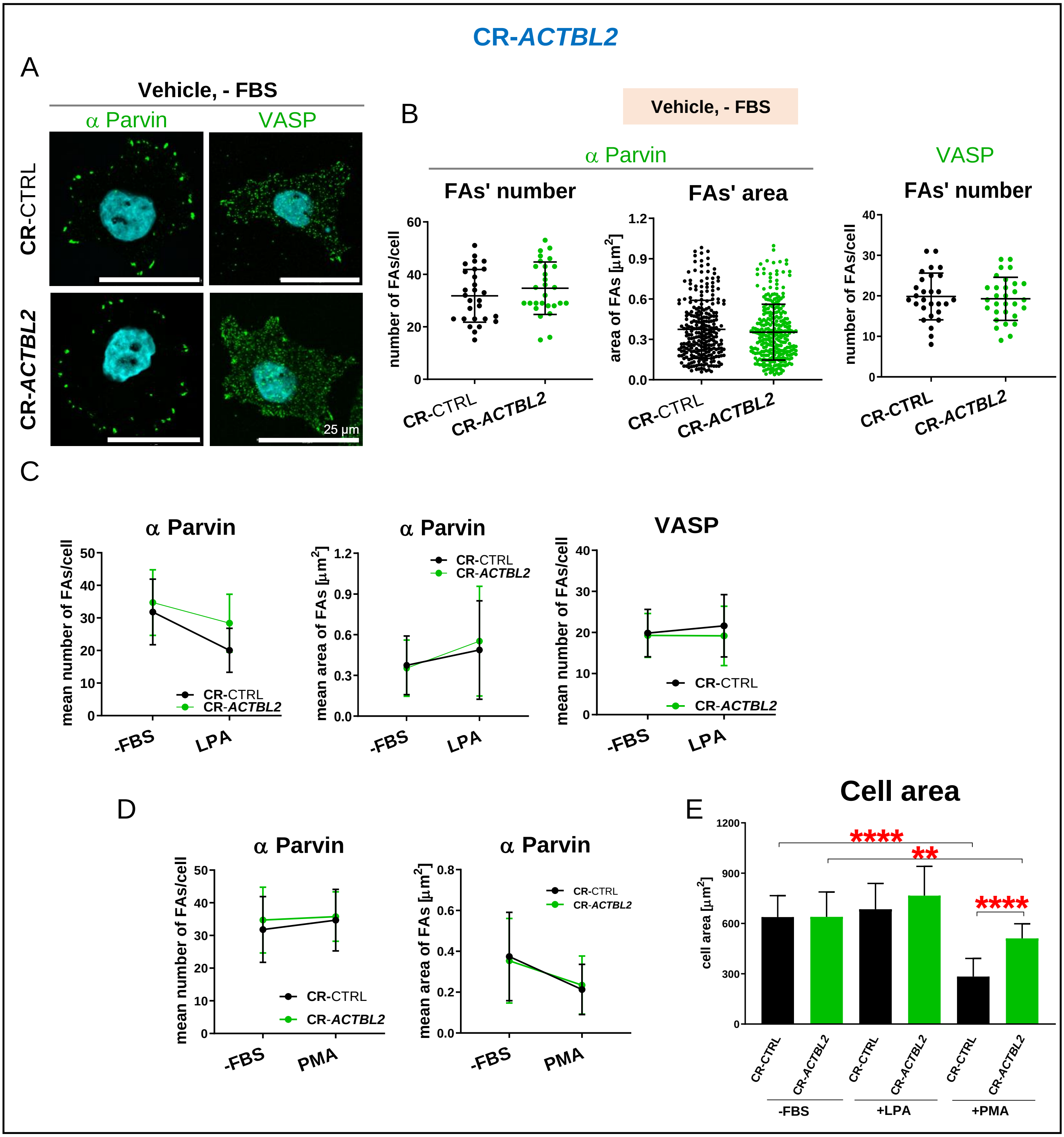

Fig. S17. Formation of focal adhesions in CR-*ACTBL2* cells grown in medium without FBS for 24 h. (**A**) The cells were seeded on coverslips. Some of them were treated with either 1 mM LPA for 10 min. or 100 nM PMA for 5 min. 24 h later, they were fixed. The cells were stained with antibodies recognizing either α Parvin or VASP. (**B**) Based on captured photos, the FAs number and their area were estimated (n=30). (**C, D**) Slope graphs presenting trends upon LPA or PMA stimulation in the number and surface area of focal adhesion regarding starvation conditions. (**E**) Cell area surface of the cells grown in the absence of FBS or treated with either LPA or PMA was measured (n=30). Results are expressed as the mean±SD; $p \leq 0.01$ (**), $p \leq 0.0001$ (****).

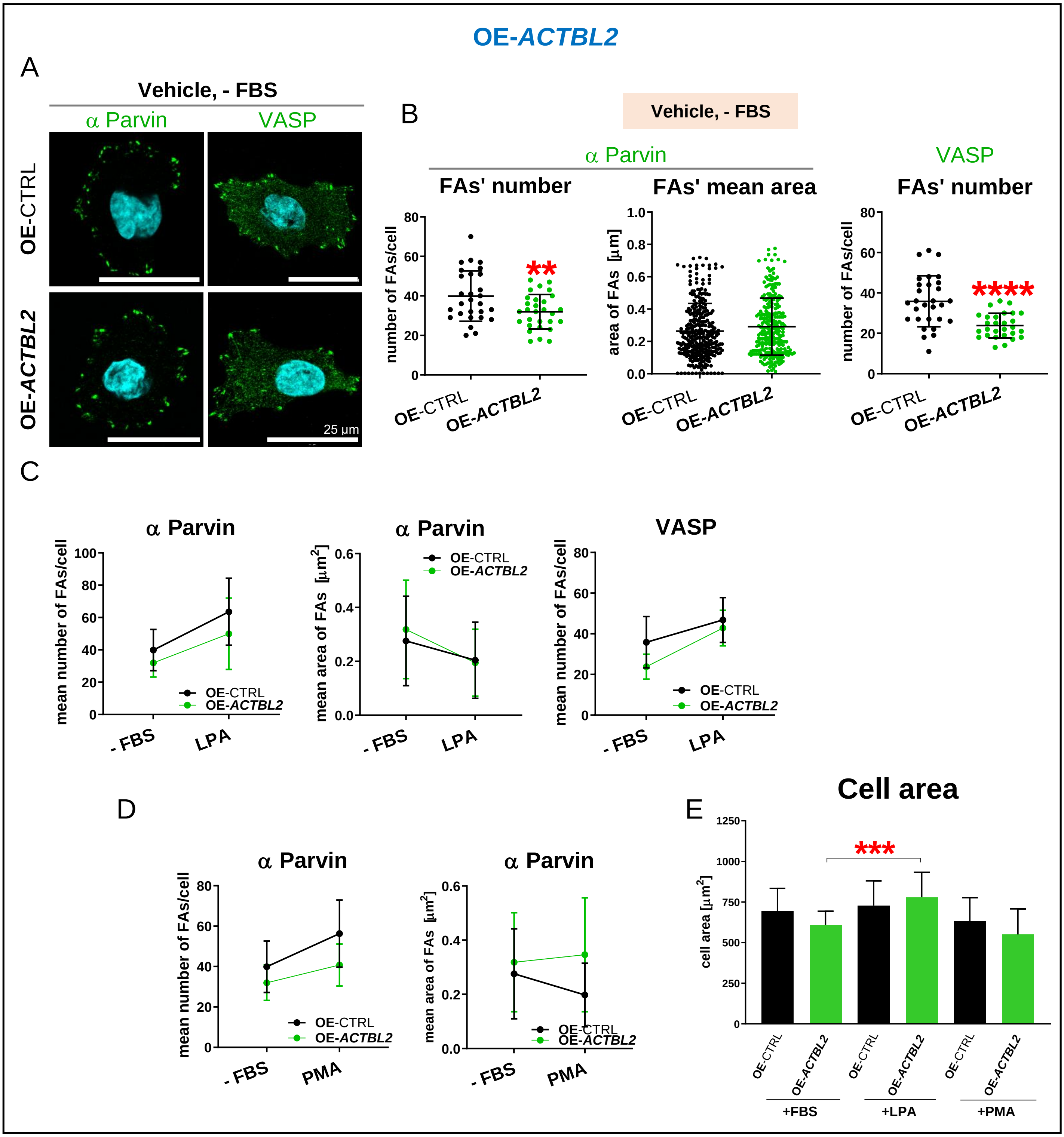

Fig. S18. Formation of focal adhesions in OE-*ACTBL2* cells grown in medium without FBS for 24 h. **(A)** Upon seeding the cells on the coverslips and treatment, some of them with either 1 mM LPA for 10 min or 100 nM PMA for 5 min, the cells were fixed and stained with antibodies directed against either α Parvin or VASP. **(B)** After taking the stained cells' photos presented in A, the FAs number and their area were measured and presented in bar charts (n=30). **(C, D)** Slope graphs presenting trends upon LPA or PMA stimulation in the number and surface area of focal adhesion. **(E)** The cell surface area of OE-CTRL and OE-*ACTBL2* cells growing it the absence of FBS or upon treatment with either LPA or PMA was measured (n=30). Results are expressed as the mean±SD; p ≤ 0.01 (**), p ≤ 0.001 (***), p ≤ 0.0001 (****).

Table S1. The number of sequences assigned to seven actin groups.

Actin group	Initial assignment based on phylogenetic grouping				Final assignment based of HMM profiles and phylogenetic trees	
	Amino acid sequences		Nucleotide sequences		Amino acid sequences	Nucleotide sequences
	IQ-TREE	MrBayes	IQ-TREE	MrBayes		
ACTB (β cytoplasmic)	107	104	57	175	105/290*	465/469*
ACTG1 (γ 1 cytoplasmic)	125	43	63	119	131/387*	63/63*
ACTA2 (α 2 smooth muscle)	26	25	235	213	26/260*	248/252*
ACTA1 (α 1 skeletal muscle)	40	68	95	149	68/318*	222/226*
ACTC1 (α cardiac muscle 1)	4	18	189	179	27/221*	375/385*
ACTG2 (γ 2 smooth muscle)	47	48	166	150	47/233*	210/216*
ACTBL2 (β like 2)	97	143	178	189	98/120*	185/187*

* The number of sequences including identical versions.

Tab. S2. Results of tests comparing tree topologies for seven groups of actins presented in Fig. S8. The table includes: log-likelihood values (logL) and their difference to the best tree (ΔlogL), marginal log-likelihood values ($logL^m$), p-values from an approximately unbiased test (AU), bootstrap probabilities calculated from all sets of scaled replicates (NP) and from one set of replicates (BP), Bayesian posterior probabilities calculated by BIC approximation (PP), p-values from Shimodaira-Hasegawa (SH) and weighted Shimodaira-Hasegawa (wSH) tests, bootstrap proportion using RELL method (bp-RELL), Expected Likelihood Weight (c-ELW) as well as Bayes factor (BF) expressed as differences in natural logarithm likelihood units from the best topology. P-values smaller than 0.05 and BF > 3 were bolded.

Program	Parameter/test	Tree topology			
		t1	t2	t3	t4
PHyML	logL	-1459.02	-1459.7	-1462.09	-1464.12
	ΔlogL	0	-0.68406	-3.0732	-5.10579
	AU	0.778	0.476	0.19	0.06
	NP	0.461	0.378	0.132	**0.03**
	BP	0.459	0.379	0.132	**0.029**
	PP	0.642	0.324	**0.03**	**0.004**
	SH	0.885	0.66	0.344	0.17
	WSH	0.893	0.638	0.311	0.209
IQ-TREE	logL	-1482.42	-1482.42	-1485.85	-1487.96
	ΔlogL	0.002	0	3.4328	5.5387
	bp-RELL	0.392	0.446	0.126	**0.0357**
	SH	0.806	1	0.377	0.217
	WSH	0.806	0.768	0.324	0.191
	c-ELW	0.388	0.434	0.132	**0.0455**
	AU	0.669	0.629	0.172	0.0646
MrBayes	$logL^m$	-1516.48	-1516.35	-1518.81	-1522.4
	BF	0.13	0	2.46	**6.05**

Table S3. Summary of the essential proteomic data confirming the presence of actbl2 at the protein level in human cells. Unique actbl2 peptides identified in these studies are shown in Fig. S9. References' numbers refer to the references' list in the main manuscript.

Year of publication	Type of cells	Biological relevance	Reference
**TUMOR CELLS**			
**2012**	MCF7 (breast carcinoma)	Actbl2 was ubiquitylated, as confirmed in two out of two experiments.	29
**2012**	GAMG (glioblastoma cell line)	Actbl2 specific peptide were identified in the proteome analysis of Glioblastoma.	43
**2014**	HeLa (cervical adenocarcinoma)	Actbl2 was a target for FAT10 (ubiquitin-like modifier), as confirmed in at least two experiments out of 4.	28
**2016**	Breast carcinoma	Actbl2 was shown to be one of the proteins present in the primary tumors as well as in lymph node metastases at a higher level than in healthy ductal epithelia.	31
**2016**	Glioblastoma cell lines	Actbl2 level was shown to be upregulated in glioblastoma cell lines, but it was not identified as an upregulated tumor antigen. (Cells treated with antitumor drug causing upregulation of specific peptides in the hope of providing new targets for anticancer therapy).	22
**2016**	Hepatocytes HepG2 (hepatocellular cancer cells)	Actbl2 was detected both in human hepatocytes as well as in the HepG2 cell line.	21
**2016**	Lung adenocarcinoma	Presence of actbl2 was identified in lung adenocarcinoma patient samples.	30
**2017**	HeLa (cervical adenocarcinoma)	Analysis of HeLa proteome showed actbl2 to be phosphorylated at S240.	19
**2017**	Human colorectal cancer tissue specimens and the comparable regions of adjacent normal mucosa	Actbl2 was found to be upregulated in cancer tissue of patients suffering from colorectal cancer tissue. Actbl2 was as well present in healthy tissue.	25
**2017**	Anal cancer carcinoma	Actbl2 was identified to be present in different parts of the anal canal in anal cancer carcinoma samples. No marked actbl2 upregulation was noted in any of the regions.	39
**2017**	HeLa (cervical adenocarcinoma) UNO2	Actbl2 was shown to be SUMOylated. No other additional PTMs were detected along with the SUMOylation.	20
**2017**	Human cancer cell lines	Unique actbl2 peptides (at least one per cell line) were identified in four human cell lines: K-562, MV-4-11, SK-N-BE(2), COLO 205.	37
**2017**	Human kidney cancer	Actbl2 presence was detected in kidney cancer. Unique actbl2 peptide was not phosphorylated according to this study.	32
**2017**	NSCLC Non-small cell lung carcinoma	Actbl2 was shown to be present in NSCLC cells. The phosphorylation did not occur at any of the unique peptides in the protein.	35

Table S3. Continued

Year of publication	Type of cells	Biological relevance	Reference
**TUMOR AND NORMAL CELLS**			
**2018**	Pancreatic cancer	Actbl2 specific peptide was recognized in pancreatic cell cancer.	42
**2011**	U2OS (osteosarcoma) HEK293T (human kidney embryonic cells)	A large-scale study of ubiquitylated protein sites. Identified unique actbl2 peptide, according to this study, was not ubiquitylated.	24
**2011**	HCT116 (colorectal carcinoma) HEK293T (human kidney embryonic cells)	A large-scale study of ubiquitylated protein sites. Actbl2 was ubiquitylated at Lys^{51} within a unique actbl2 peptide (a different one than those identified by Danielsen et al. [24]).	26
**NORMAL CELLS**			
**2016**	BJ (human normal foreskin fibroblasts) NIH3T3 (murine fibroblasts) HEK293T (human kidney embryonic cells)	Actbl2 was among seven proteins interacting with FGF1.	27
**2016**	HUVEC (human umbilical vein endothelial cells) NHDF (normal human dermal fibroblasts)	The level of actbl2 differed in HUVEC cells and NHDF cells with and without inflammatory activation.	34
**2016**	Human stem cells	Expression of actbl2 was higher in MSC than in ESC, the implication in adhesion, and developmental processes. Unique actbl2 peptides were identified in 3 out of 3 experiments.	38
**2017**	Healthy human tissue	Identification of SAAV (single amino acid variants) in one of the unique actbl2 peptides (VAPDEHPILLTEAPLNPK) in the human cortex, spinal cord, fetal brain, testis, and sperm (H102->Y102).	23
**2017**	Human heart tissue	Actbl2 level was varying across the heart regions. Actbl2 was downregulated in patients with atrial defibrillation when compared to healthy controls.	36
**2017**	Immune cells	Actbl2 specific peptides were recognized in immune cells, either after activation or in a steady state.	41
**2019**	Healthy human tissue	Actbl2 was identified to be present in the human liver, kidney, colon, and bladder. Actbl2 was as well shown to be tissue enhanced in the heart.	33

Table S4. Summary of the cloning strategies used to generate vectors used in the study.

Vector	Primers' sequences	Description
**pLVX-hPGK-Puro**	5'atcaccgacctctctccccaagagctcgtttagtga3'<br>5'tttgtcagacatggtgaattcaccggaaatagatcc3'<br>used when as a template served pLKO.1-puro plasmid coding hPGK<br><br>5'atcaccgacctctctccccaagagctcgtttagtga3'<br>5'tttgtcagacatggtgaattcaccggaaatagatcc3'<br>used when as e template served a DNA fragment coding for the spacer between hPGK and the sequence coding for POI:<br>5'agagctcgtttagtgaaccgtcagatcgcctggagacgccatccacgctgttttgacctccatagaagacaccgactctactagaggatctatttccggtg3'	pLVX-hPGK-Puro was obtained on the basis of pLVX-IRES-puro-Tb4 plasmid described elsewhere [3]. Here, the CMV promoter was substituted with hPGK by using *Cla*I and *Eco*RI restriction enzymes. Two PCR products were simultaneously cloned into the cut plasmid using NEBuilder® HiFi DNA Assembly Cloning Kit (New England BioLabs Inc.).
**pLVX-hPGK-Puro-actbl2 3'UTR**	5'tagaggatctatttccggtgaattcaccatgactgacaatgagctg3'<br>5'taggggggggggagggagaggggcgggatccatacaagtatgaagcaattttaatgtactgaaag3'	pLVX-hPGK-Puro-actbl2 3'UTR plasmid was prepared based on pLVX-hPGK-Puro. The actbl2 coding sequence obtained from A375 cells was cloned into the plasmid with the use of *Eco*RI and *Bam*HI restriction enzymes using NEBuilder® HiFi DNA Assembly Cloning Kit (New England BioLabs Inc.).
**p3xHA-actbl2-3'UTR**	5'aaactcgagttatgactgacaatgagctgtc 3'<br>5'aaatctagaatacaagtatgaagcaat3'	3xHA-C1-actbl2-3'UTR was obtained using *Xho*I and *Xba*I restriction enzymes.
**p3xHA-β actin-3'UTR**	5'gttatggatgatgatatcgccgcg3'<br>5'gctaaggtgtgcacttttattcaac3'	3xHA-C1-β actin-3'UTR was obtained using *Xho*I and *Xba*I restriction enzymes.
**p3xHA-γ actin-3'UTR**	5'gttatggaagaagagatcgccgc 3'<br>5'gggttacggcagcacttttatttt3'	3xHA-C1-γ actin-3'UTR was obtained using *Xho*I and *Xba*I restriction enzymes.
**p3xHA-amplified fragment of allele coding for actbl2**	5'gatctcgagctcaagcttcgaattcgaaagcatttctggagtgttttag<br>5'cgcggtaccgtcgactgcagaattcatacatggctggtgtgttg	Amplified on the basis of isolated gDNA fragment of allele coding for actbl2 was cloned into pAcGFP-C1 vector linearized with *Eco*RI. We used here NEBuilder® HiFi DNA Assembly Cloning Kit (New England BioLabs Inc.).

Table S5. List of primers used in the study for other purposes than cloning.

Primer	Sequence	Amplicon size (nt)	Tm (°C)
**qPCR**			
*ACTBL2*_f<br>*ACTBL2*_r	5'→gacaatgagctgtctgcc<br>5'→cgtagcagtccttctggc	157	60
*ACTB*_f<br>*ACTB*_r	5'→tttcttgacaaaacctaacttgcgc<br>5'→attgtgaactttgggggatgctct	175	61
*ACTG1*_f<br>*ACTG1*_r	5'→gcattgccgacaggatgcag<br>5'→atgcagcaaatgctacgcatctg	239	64
*HPRT1*_f<br>*HPRT1*_r	5'→gaccagtcaacaggggacat<br>5'→gcttgcgaccttgaccatct	165	60
**gDNA analysis (Fig. S12)**			
*ACTBL2*_f<br>*ACTBL2*_r	5'→gacaatgagctgtctgcc<br>5'→cgtagcagtccttctggc	157	60

Table S6. List of antibodies and fluorescently labeled reagents used in the study.

Antibody	Company	ICC	WB
mouse anti-β actin IgG_1 (#A5441)	Sigma-Aldrich	-	1:20000
mouse anti-γ actin IgG_1 (#A8481)	Sigma-Aldrich	-	1:20000
rabbit anti-total actin C11 (#A2066)	Sigma-Aldrich	-	1:1000
mouse anti-total actin AC40 (#A3853)	Sigma-Aldrich	-	1:1000
anti-actbl2 (#OALA08260)	Aviva Systems Biology	-	1:200
goat anti-HA (#NB600-362) (Fig. 5A)	Novus Biologicals	1:100	-
rabbit anti-HA (#51064-2-AP) (STED, Fig. 5B)	Proteintech	1:100	-
rabbit anti VASP (#3132)	Cell Signaling	1:400	-
rabbit anti-α Parvin (#8190)	Cell Signaling	1:200	-
HRP-conjugated anti-rabbit (#7074)	Cell Signaling	-	1:4000
HRP-conjugated anti-mouse (#7076)	Cell Signaling	-	1:4000
donkey anti-goat-Alexa Fluor® 488 (#A-11055)	Invitrogen	1:200	-
donkey anti-rabbit-Alexa Fluor® 488 (#A-21206)	Invitrogen	1:200	-
donkey anti-rabbit-Alexa Fluor® 647 (STED, Fig. 7B) (#A-31573)	Invitrogen	1:200	
DRAQ5	Invitrogen	1:100	-
Phalloidin CruzFluor™ 350	Santa Cruz Biotechnology Inc.	1:1000	-
Phalloidin Alexa Fluor® 488	Invitrogen	1:100	-
Phalloidin Alexa Fluor® 568	Invitrogen	1:100	-
Phalloidin Abberior Star Red (STED, Fig. 8 and 9)	Abberior	1:100	-
DNase I Alexa Fluor® 549	Invitrogen	1:100	-
Hoechst 33342	Invitrogen	1:1000	-

References

1. Katoh, K. & Standley, D. M. MAFFT multiple sequence alignment software version 7: improvements in performance and usability. *Mol. Biol. Evol.* **30**, 772–80 (2013).
2. dos Remedios, C. G. & Moens, P. D. Actin and the actomyosin interface: a review. *Biochim. Biophys. Acta* **1228**, 99–124 (1995).
3. Makowiecka, A. *et al.* Thymosin β4 regulates focal adhesion formation in human melanoma cells and affects their migration and invasion. *Front. Cell Dev. Biol.* **7**, 304 (2019).
